# Photonic Chip Based on Ultrafast Laser-Induced Reversible Phase Change for Convolutional Neural Network

**DOI:** 10.1007/s40820-025-01693-5

**Published:** 2025-03-11

**Authors:** Jiawang Xie, Jianfeng Yan, Haoze Han, Yuzhi Zhao, Ma Luo, Jiaqun Li, Heng Guo, Ming Qiao

**Affiliations:** 1 State Key Laboratory of Tribology in Advanced Equipment, Tsinghua University, Beijing, 100084 People’s Republic of China; 2 Department of Mechanical Engineering, Tsinghua University, Beijing, 100084 People’s Republic of China

**Keywords:** Photonic chip, Ultrafast laser, Phase change, Convolutional neural network

## Abstract

Photonic computing has emerged as a promising technology for the ever-increasing computational demands of machine learning and artificial intelligence. Due to the advantages in computing speed, integrated photonic chips have attracted wide research attention on performing convolutional neural network algorithm. Programmable photonic chips are vital for achieving practical applications of photonic computing. Herein, a programmable photonic chip based on ultrafast laser-induced phase change is fabricated for photonic computing. Through designing the ultrafast laser pulses, the Sb film integrated into photonic waveguides can be reversibly switched between crystalline and amorphous phase, resulting in a large contrast in refractive index and extinction coefficient. As a consequence, the light transmission of waveguides can be switched between write and erase states. To determine the phase change time, the transient laser-induced phase change dynamics of Sb film are revealed at atomic scale, and the time-resolved transient reflectivity is measured. Based on the integrated photonic chip, photonic convolutional neural networks are built to implement machine learning algorithm, and images recognition task is achieved. This work paves a route for fabricating programmable photonic chips by designed ultrafast laser, which will facilitate the application of photonic computing in artificial intelligence.

**Supplementary Information:**

The online version contains supplementary material available at 10.1007/s40820-025-01693-5.

## Highlights

- Programmable photonic chips based on ultrafast laser-induced phase change is fabricated for photonic computing.
- Based on the integrated photonic chip, photonic convolutional neural networks are built to implement machine learning algorithm, and images recognition task is achieved.
- The transient laser-induced phase change dynamics of Sb film are revealed at atomic scale, and the phase change time is measured.

**Supplementary Information:**

The online version contains supplementary material available at 10.1007/s40820-025-01693-5.

## Graphical abstract

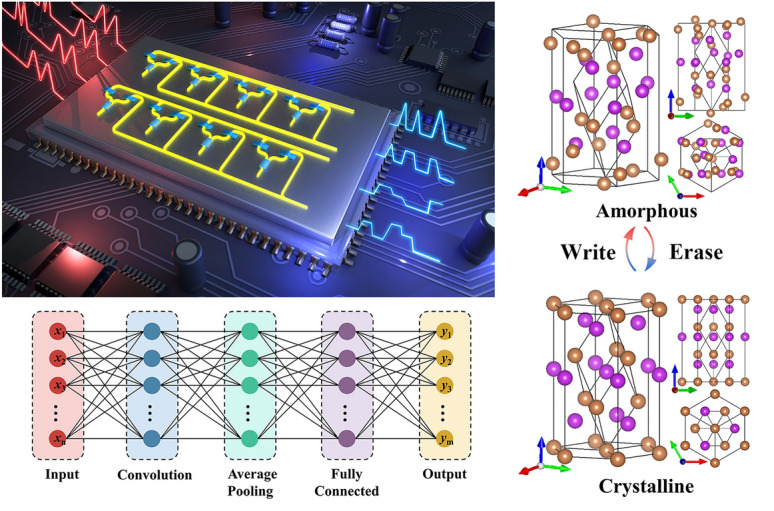

## Introduction

With the rapid increase in dataset size and computational cost, computing hardware with high calculation speed is strongly desired for the development of machine learning and artificial intelligence [1–7]. Several technologies have been proposed for the next-generation computing accelerator, such as quantum computing [8, 9], neuromorphic computing [10–13], and photonic computing [14–18]. Integrated neuromorphic photonic networks have emerged as a promising hardware accelerator for complex matrix–vector multiplication and convolution operation, which are the fundamental operation in artificial neural networks [19–21]. Compared with electronic devices, integrated photonic devices show advantages in computing speed, throughput, bandwidth density, and power efficiency [22–24]. As the fabrication of silicon photonic integrated circuits (PICs) is compatible with CMOS technology, silicon PICs show a prospective application to establish compact computing units in neural photonic networks [25]. Several silicon PICs architectures have been proposed to realize integrated optical neural networks, such as cascades of multiple Mach–Zehnder interferometers [26, 27], micro-ring resonator-based wavelength division multiplexing [28], waveguide mode converter-based convolution [29], and diffractive neural networks [30].

To realize optical computing with PICs, programmable photonic components are essential building blocks [26, 31]. Electric signals have been used to program the PICs based on thermo-optic effect and free carrier dispersion effects. In general, PICs units are integrated with micro-heaters, which are controlled by electric circuits [14, 30]. By switching the electric current, the temperature of PICs units can be controlled. Owing to thermos-optic effect, the refractive index of silicon waveguide is programmed by turning the temperature [32, 33]. As the thermal conductivity of silicon is poor, the switching speed is limited, and requires large power consumption. Free carrier dispersion effect is another method for programming PICs. By modulating the voltage on waveguide, free carrier concentration (electrons and holes) can be controlled, which can change the refractive index of silicon [34–36]. Both thermo-optic and free carrier dispersion effects are volatile and require supplemental power source to maintain the programmed state, which limit their application in integrated compact devices. Phase change materials (PCMs) have recently emerged as a leading candidate to build nonvolatile programmable PICs without power source [22, 29]. PCMs can be switched between two solid phases, the amorphous and crystalline phases, which show a difference in the complex refractive index. Compared with thermo-optic and free carrier dispersion effects, PCMs show larger change in refractive index and faster switching speed, which have attracted the attention in the fabrication of programmable PICs. For example, by incorporating PCMs with silicon PICs, Majumdar et al. have achieved broadband waveguide switching and multilevel phase modulator based on micro-ring resonator [23]. As the optical modulation of PCMs is nonvolatile, sustaining power supply is not required to retain the programmed state, which reduces the power consumption and complexity of PICs [37, 38].

Various PCMs have been applied in programmable PICs, such as Ge_2_Sb_2_Te_5_ (GST) [39], Sb_2_Se_3_ [40, 41], and Sc_0.2_Sb_2_Te_3_ (SST) [42]. As a conventional PCM, GST has been widely explored for both optical and electrical applications, and includes reflective displays, tunable emitters, and reconfigurable meta-surface [43–45]. The antimony-based chalcogenides are a new family of low-loss PCMs for photonic applications, including Sb_2_Se_3_ [40], Sb_2_S_3_ [46], and SST [42], which show negligible absorption in either phase over the telecommunications wavelength [46]. To switch the solid phase of PCMs, it is essential to control the heating and cooling process of PCMs. Generally, a rapid quenching process is required to form amorphous phase, and a slow cooling process is needed for crystallization [37]. Ultrafast laser pulse can provide a rapid heating and cooling process, due to the nature of ultrashort pulse duration and high peak intensity [47, 48]. Upon ultrafast laser irradiation, the laser energy is absorbed by electron system, and transferred to lattice system and surrounding medium [49, 50]. The ultrafast heating and cooling process provides a route to reversibly switch the PCMs between amorphous and crystalline states [51]. A short laser pulse with high fluence can heat the PCMs above melting point, following a rapid quenching process of molten materials. The amorphous phase is formed under the large quenching rate. On the other hand, a laser pulse with small fluence can be used for crystallization, which slightly excites the materials to overcome the crystallization barrier. By designing the ultrafast laser fluence and pulses trains, the heating and cooling rates can be modulated, and the phase of PCMs can be controlled. Recently, the thin films of antimony (Sb) with thickness of several nanometers have been reported that can be switched between amorphous and crystalline states [52, 53]. As a monatomic PCM, Sb thin film shows advantages in cycling endurance, switching speed, and phase stability, which is limited by the compositional variation problem of ternary alloy and chalcogenides materials [53, 54]. Several studies have paid attention to the switching of Sb film; however, integrated photonic chips still need to be fabricated for actual applications. Moreover, it is complex for the ultrafast laser-induced phase change of Sb thin film because the underlying dynamics remain elusive, and the response time still needs to be explored. With the aim of building programmable photonic chips, the laser-induced phase change mechanism and corresponding response dynamics of Sb film should be revealed.

In this work, we propose a photonic chip based on phase change Sb film for photonic computing. The laser-induced amorphization of Sb film is uncovered at atomic scale and the response time is measured. By designing the ultrafast laser pulse trains and energy deposition rate, the thermal annealing and quenching process of Sb film can be controlled, and the film is switched reversibly to crystalline or amorphous phase. The optical properties and lattice structure of crystalline and amorphous phase of Sb film are analyzed by optical spectroscopy and electron microscopy. By switching the phase of Sb film, the transmission of silicon waveguide is set to desired state, and an integrated photonic chip for convolutional network is built based on the programmable waveguides. An images recognition task is implemented on the integrated photonic chips, which demonstrate the practical applications in photonic computing.

## Experimental Section

### Materials Preparation

The Sb thin films were deposited on a silicon wafer by direct-current (DC) magnetron sputtering with Sb target. The background pressure was less than 1.0 × 10^–4^ Pa, and the deposited pressure was 0.7 Pa under argon atmosphere. The deposited power and bias voltage were 30 W and 100 V, respectively. The deposited rate was 20 nm min^−1^. The thickness of as-deposited films was measured by a step profiler (Dektak XT, Bruker), as shown in Fig. S4.The samples for TEM characterization were scraped from the substrate and dropped on copper grids. The as-deposited samples were amorphous Sb film, and the crystalline films were obtained by thermal annealing the as-deposited films on a hot-plate at 220 °C for 10 min.

### Materials Characterization

The Raman spectra of Sb film were measured by LabRAM system (Horiba) using a 532-nm laser as pump source with a 100 × objective lens, and an 1800 grating. The refractive index and extinction coefficient were obtained from spectroscopic ellipsometer (Uvisel plus, Horiba). All measurements were conducted in air, at a 70° incidence angle, and the data were collected in the spectra range of 300 to 1700 nm. The measurement result was fitting using the built-in software, with the Tauc-Lorent2oscillator model. Reflection spectra of Sb film were measured using ultraviolet–visible–near-infrared (UV–VIS-NIR) spectroscopy (L950, PerkinElmer). The simulated reflection spectra were calculated by a house-built script based on the transfer matrix algorithm. The surface structure of the laser irradiated films was characterized by a scanning electron microscope (Zeiss Gemini) and optical microscopy. The lattice structure and selected area electron diffraction (SAED) of Sb films were characterized by a transmission electron microscope (JEM-2100F, JEOL).

### Ultrafast Laser-Induced Phase Change

The laser-induced phase change experiments were performed on Sb thin film with thickness of 5 nm. A Ti:sapphire femtosecond laser source was used to switch the phase of Sb film. The laser used for experiments has a wavelength of 800 nm and a pulse duration of 35 fs. The laser beam was focused by a 10 × objective lens, which gave a Gaussian focused spot with diameter of 5.1 μm (at 1/e^2^). A white LED was used for illumination and the image of sample was captured by a CCD camera. A neutral density filter was used to attenuate the laser pulse energy Ep. The relative position of the sample and laser spot was adjusted by a positioning stage. A single-pulse femtosecond laser was used to amorphize the Sb film, while a consecutive pulses train with high repetition rate was used to recrystallize the sample. The scan speed for amorphization was 100 μm s^−1^, and 200 μm s^−1^ for crystallization.

### Photonic Chips Fabrication

The photonic chips were fabricated on the silicon-on-insulator (SOI) wafers with a 220-nm silicon top layer and a 2 μm buried oxide. The wafer was cleaned with acetone and deionized water firstly, and a negative e-beam resist was coated on the wafer using spin coating method. The patterns of waveguide were exposed using e-beam lithography and developed with acetone. The waveguide was then dry etched with a depth of 220 nm. The second round of e-beam lithography patterning was performed on the fabricated waveguide to open windows (10 μm width and 20 μm length) for Sb film deposition. The thin Sb film was then deposited on the waveguide following the magnetron sputtering procedure, and then, the e-beam resist was then developed with acetone.

### Transient Reflectivity Measurement

A pump–probe imaging technique was used to measure the time-resolved response dynamics after laser excitation. Pulses generated from Ti:sapphire laser systems were split into pump and probe pulses by a beam splitter. The pump pulse was focused on the Sb film with a lens (focal length of 100 mm) at a 45° incident angle. The probe pulse was guided through a delay line, and focused on the sample by a 10 × objective lens at normal incident. The delay time between pump pulses and probe pulses was adjusted by a one-dimensional positioning stage. After the sample irradiated by pump pulse, the probe pulse illuminated on the excited area, and the reflected light was collected by a tube lens. The reflected light was focused on a charge-coupled-device camera, and the intensity distribution was recorded. The images with and without pump pulses were recorded. The normalized reflectivity change was calculated as Δ*R*/*R*_0_ = (*R*-*R*_0_)/*R*_0_, where *R* and *R*_0_ are the intensities of reflect probe pulse with and without pump pulse.

### Molecular Dynamics Simulation

The laser-induced amorphization process was modeled using a method coupling two-temperature model and molecular dynamics (TTM–MD) [49, 50]. The dimensions of TTM–MD simulation domain were 5 × 100 × 100 nm^3^, which consists of ≈1.8 million Sb atoms. Free boundary condition was applied in *x* dimension, whereas periodic boundary condition was applied in *y* and *z* dimension. Time step for MD simulation was 1 fs and the mesh resolution for FDM calculation was 1 nm. The interatomic interaction between Sb atoms was calculated by the embedded atom method (EAM) potential [51]. The thermal parameters of Sb electron were calculated by density functional theory as implemented in ABINIT. The detail about the simulation setup is provided in Supporting Information.

### Training of Convolutional Neural Network

The convolutional neural network was built and trained with a back-propagation algorithm using the gradient descent method. The network consists of an input layer, a convolutional layer, an average pooling layer, a fully connected layer, and an output layer. The nonlinear function ReLU was applied to the convolutional layer, and sigmoid function was applied to the fully connected layer. The training set consists of 6742 images of the handwritten number “1” and 5958 images of the handwritten number “2” from the MNIST database. The epoch number of the training was 3000. The training results of all matrices and bias are provided in Supporting Information.

## Results and Discussion

### Photonic Chips Based on Ultrafast Laser-Induced Phase Change

**Fig. 1:**
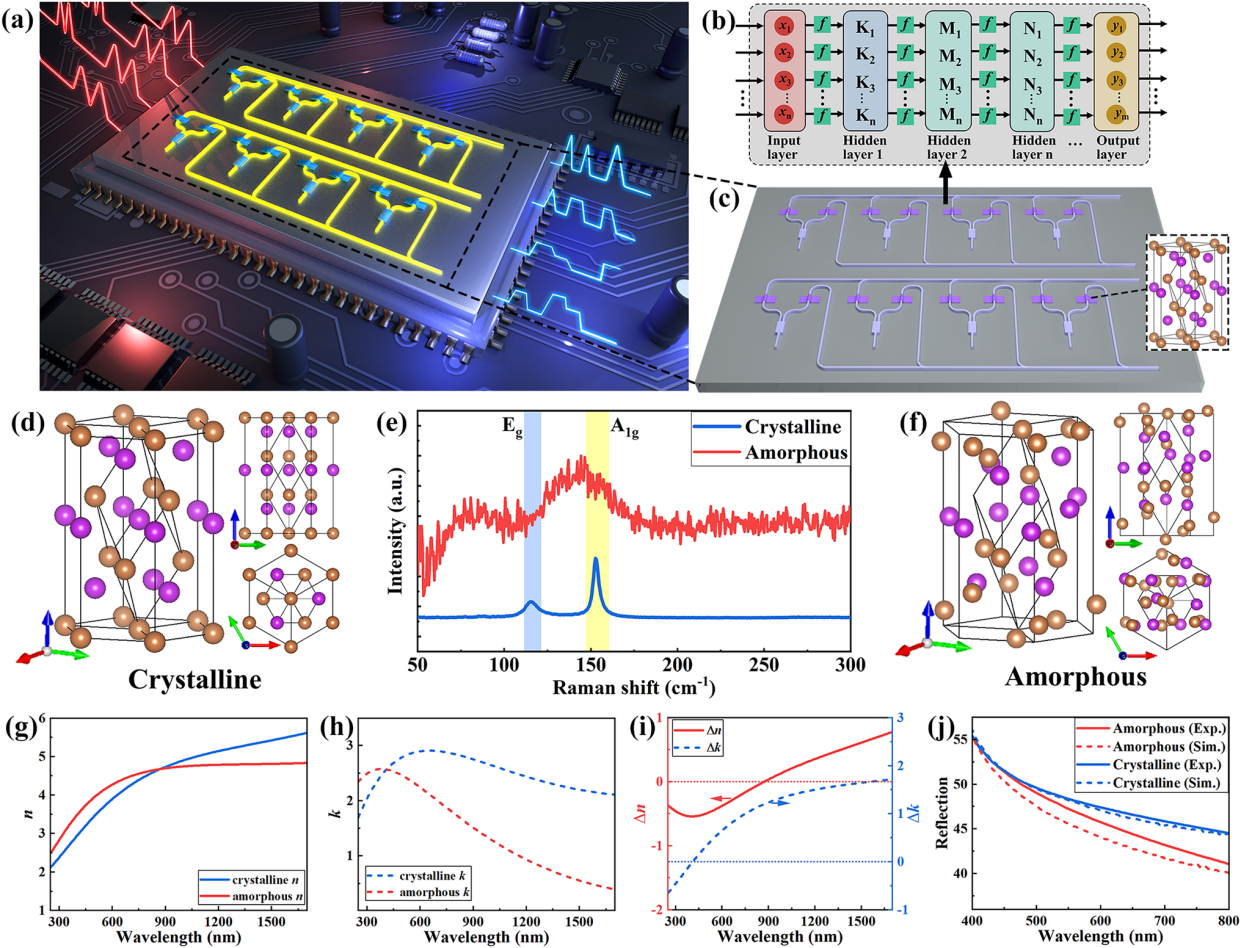
Concept of photonic chip based on ultrafast laser-induced phase change. **a** Schematic diagram of photonic chips. **b** Representative architecture of multilayer neural networks. **c** Schematics of photonic convolutional network based on Sb film. The purple blocks illustrate PCM Sb film. **d** Illustration of crystalline structure of Sb. **e** Raman spectra of crystalline (blue) and amorphous (red) Sb film. **f** Illustration of amorphous structure of Sb. **g** Spectra of refractive index of crystalline (blue) and amorphous (red) Sb film. **h** Spectra of extinction coefficient of crystalline (blue) and amorphous (red) Sb film. **i** Contrast of refractive index (red) and extinction coefficient (blue) of Sb film. **j** Reflection spectra of crystalline (blue) and amorphous (red) Sb film. The solid lines were experiment data, and the dashed lines were simulation results

Figure 1a illustrates the concept of programmable photonic chips, which can be programmed by ultrafast laser pulse. The input optical signals are modulated by the waveguides, and neural network algorithm is implemented with designed photonic networks. The typical architecture of multilayer convolutional neural network algorithm is presented in Fig. 1b, which consists of an input layer, an output layer, and several hidden layers. Convolution transformation is a fundamental matrix multiplication in hidden layers, which can be implemented in the optical field using PICs. Figure 1c presents a photonic convolution network based on Sb thin film. By switching the phase of Sb film with ultrafast laser pulse, the weight matrices of hidden layer are programmed on PICs. The atomic structure of crystalline Sb film is shown in Fig. 1d, which belongs to rhombohedral lattice system with space group R $\stackrel{\text{-}}{3}$ m. The amorphous Sb has a disorder arrangement of atoms, as shown in Fig. 1f. The Raman spectra of crystalline and amorphous Sb film (with a thickness of 5 nm) have been measured and presented in Fig. 1e. The Raman peaks of E_g_ (~ 114 cm^−1^) and A_1g_ (~ 151 cm^−1^) are found in crystalline Sb, which are related to the in-plane and out-of-plane vibrational modes of Sb [58]. The red line in Fig. 1e is the Raman spectra of amorphous Sb film. The E_g_ and A_1g_ peaks are absent in amorphous Sb film, as the atoms are in disorder arrangement and the vibrational modes of crystalline Sb are lost. The samples maintain the phase for at least 100 days at atmospheric environment (Fig. S5), indicating that the phase change modulation is nonvolatile. The optical properties of crystalline and amorphous Sb film were further characterized by ellipsometry and UV–visible-near-infrared light spectrophotometer. Figure 1g, h presents the refractive index (*n*) and extinction coefficient (*k*) of crystalline and amorphous Sb film. Significant contrast of *n* and *k* between crystalline and amorphous Sb film is found, and the contrast of refractive index (Δ*n*) and extinction coefficient (Δ*k*) is plotted in Fig. 1i. In telecommunication wavelength bands (from 1.5 to 1.6 μm), the contrast of refractive index is ~ 0.65, and the change in extinction coefficient is ~ 1.66. The large contrast of extinction coefficient allows the application for optical amplitude modulation in PICs. Figure 1j presents the reflection spectra of crystalline and amorphous Sb film. It is showed that the reflection of Sb film decreases in the wavelength of 400 to 800 nm after amorphization. The dashed lines are the simulated spectra using the *n* and *k* values measured by ellipsometry. The simulation spectra are consistent with the experimental results. It should be noted that the Raman peaks of antimony oxide (~ 191 and ~ 255 cm^−1^) are absent in Fig. 1e, which indicates that the change of optical properties of Sb film is due to the laser-induced phase change rather than oxidation of Sb film.

### Laser-Induced Reversible Phase Change

**Fig. 2:**
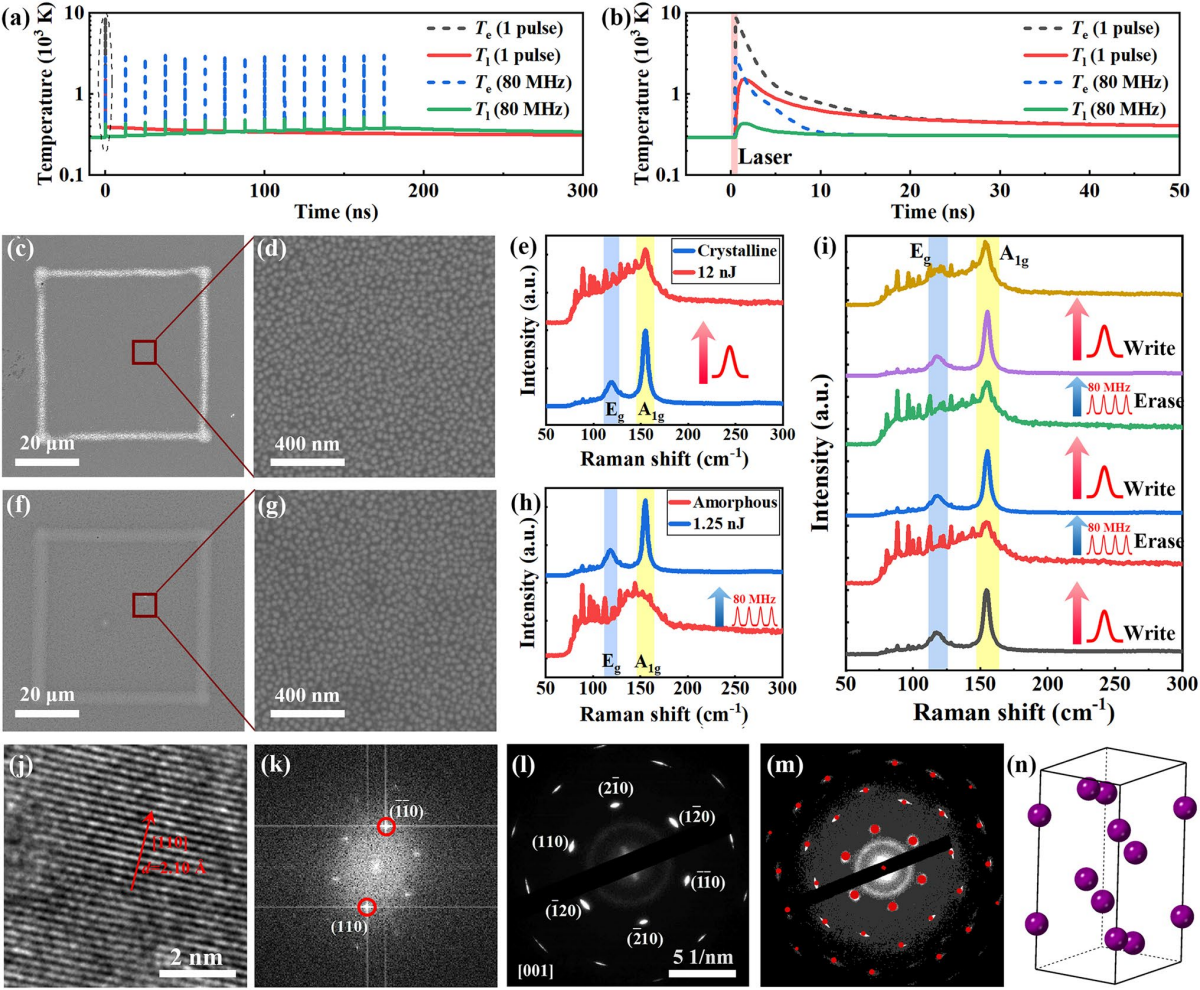
Microstructure transition analysis of laser-induced phase change. **a** Electron temperature (dashed lines) and lattice temperature (solid lines) evolution of Sb film irradiated by single pulse with *E*_p_ = 12 nJ, and multi-pulse with *E*_p_ = 1.25 nJ and repetition rate of 80 MHz. **b** Enlarged view of the dashed circle in h, showing the temperature evolution detail after one pulse irradiation. **c, d** SEM image of crystalline Sb film irradiated by single-pulse laser with* E*_p_ = 12 nJ. **e** Raman spectra of crystalline Sb film before (blue) and after (red) laser irradiation. **f, g** SEM image of amorphous Sb film irradiated by pulse train with *E*_p_ = 1.25 nJ. **h** Raman spectra of amorphous Sb film before (red) and after (blue) laser irradiation. **i** Raman spectra of Sb film after multi “write-erase” cycles. **j** The TEM image of laser-induced crystalline Sb film. **k** Fourier transform of TEM image, showing reflections (110). **l** Electron diffraction pattern of the crystalline Sb film. **m** Comparison of measured and simulated (red dots) electron diffraction pattern. **n** Unit cell of the rhombohedral crystalline structure of Sb

The reversible laser-induced phase change of Sb thin film was explored using ultrafast laser pulses. Figure 2 presents the microstructure and Raman spectra of laser-induced crystallization samples and amorphization samples. The temperature evolution of Sb film irradiated by single pulse and high repetition rate pulse train is calculated and presented in Fig. 2a, b. Owing to the high peak energy and ultrashort pulse duration, single-pulse irradiation generates a large cooling rate, which is necessary for the formation of amorphous phase. The multi-pulse irradiation led to a small temperature increase, as the pulse energy is smaller than single pulse. The heat accumulation effect of multi-pulse irradiation offers a moderate heating and cooling process. Therefore, a pulse train with a high repetition rate and a small pulse energy was used to crystallize the amorphous film.

The laser-induced amorphous Sb film is shown in Fig. 2c. The region inside the box is irradiated by a single femtosecond laser pulse with pulse energy* E*_p_ = 12 nJ. The enlarged image in Fig. 2d suggests that there is no damage to the film after laser irradiation. The Raman spectra of the film before and after laser irradiation are presented in Fig. 2e. The Raman spectra confirm that the crystalline Sb film (blue line) changed to amorphous Sb film (red line) after irradiated by a single femtosecond laser pulse. Single pulse with various pulse energy was used to amorphize the Sb film, and the corresponding Raman spectra are provided in Supporting Information (Fig. S6). It is shown that the crystalline sample switches to amorphous phase when the pulse energy is larger than 10 nJ. The optical and SEM images (Figs. S7 and S8**)** show that the film is damaged when the pulse energy is larger than 16 nJ. Figure 2f is the amorphous Sb film irradiated by a pulse train with a repetition rate of 80 MHz and *E*_p_ = 1.25 nJ (energy of each pulse in the train). The Raman spectra of the sample before and after crystallization are shown in Fig. 2h. It is confirmed that the amorphous film (red line) switches to crystalline film (blue line) after irradiated by a high repetition rate pulse train. The enlarged view in Fig. 2g shows that the film is not damaged by the laser pulse. More laser-induced crystallization experiments were carried out and the results are presented in Supporting Information (Fig. S6). It is shown that the amorphous film switched to crystalline film when the pulse energy is larger than 0.25 nJ, and damaged when the pulse energy is larger than 2.00 nJ (Figs. S9 and S10).

Reversible laser-induced phase change experiments were carried out. The crystalline film was switched to amorphous film by a single pulse with *E*_p_ = 12 nJ firstly, which was named “write” process. The amorphous film was then switched to crystalline film by a pulse train with *E*_p_ = 1.25 nJ, which was named “erase” process. Three “write-erase” cycles were performed on the same film and the results are presented in Fig. 2i. It is shown that the sample switched to amorphous state after irradiated by “write” pulse, and switched to crystalline state after irradiated by “erase” pulse. The SEM images (Fig. S11) of the films after multiple scans confirm that the films keep the initial structure. In order to study the durability of the film, 100 switching cycles were performed, and no obvious change is found (Figs. S12 and S13), which suggests that the phase change process is durable.

To further identify the crystal structure of Sb film, transmission electron microscopy (TEM) and selected area electron diffraction (SAED) were performed. The amorphous Sb film was crystallized by ultrafast laser pulse, then scraped from the substrate and dropped on the copper grids. The energy-dispersive spectrometer mapping confirms the existence of Sb film, as shown in Supporting Information (Fig. S14). Figure 2j shows the lattice fringes of crystalline Sb film, and the corresponding Fourier transform image is shown in Fig. 2k. The spacing of the lattice fringes is measured as 2.10 Å using the Fourier transform spots in Fig. 2k. The measured spacing is consistent with the (110) plane of Sb crystal. The SAED pattern in Fig. 2l shows a hexagonal lattice structure. By comparing the measured SAED pattern with the simulated pattern (red dots in Fig. 2m**)**, it is confirmed that the crystalline Sb has a rhombohedral unit cell with space group R $\overline{3 }$ m, as the schematic images in Fig. 2n. The zone axis of the measured sample is [001], as confirmed by the SAED patterns.

**Fig. 3:**
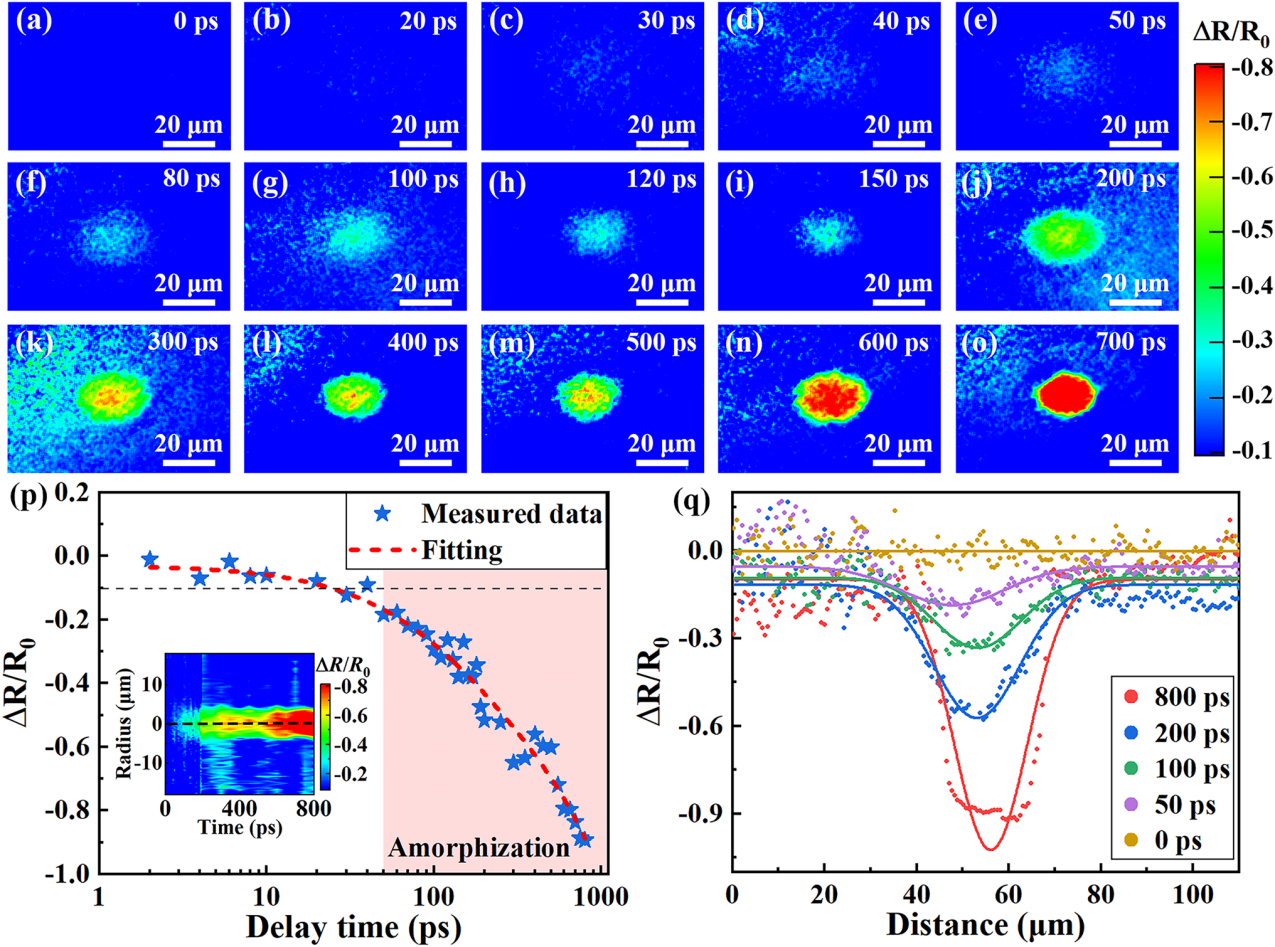
Time-resolved dynamics of transient reflectivity during laser-induced amorphization. **a–o** 2D mapping of the transient reflectivity change of Sb film at delay time from 0 to 800 ps after laser irradiation. **p** Transient reflectivity change of the spot center versus delay time, the inset is the 2D mapping of transient reflectivity change versus delay time and position. **q** Spatial transient reflectivity profile along the long axis of the focal area, the solid lines are Gaussian fitting results

The response dynamics of laser-induced amorphization were explored by time-resolved pump–probe imaging technique. Figure 3a–o shows the transient reflectivity of Sb film at various delay times after laser excitation. The normalized reflectivity changes before and after laser excitation is presented, which is defined as Δ*R*/*R*_0_ = (*R*-*R*_0_)/*R*_0_. *R* and *R*_0_ are the intensities of reflect probe pulse with and without pump laser excitation, respectively. Figure 3e, f shows that a blurry low reflectivity region (light blue region) emerged from the laser irradiated spot after pump laser excitation. The reflectivity decrease indicates the arising of amorphization, as the reflectivity of amorphous Sb film is smaller than crystalline Sb film. The reflectivity decreases gradually with the delay time, and the spot pattern become clearer, as shown in Fig. 3g–o. More 2D mapping dates of reflectivity at different delay time are shown in Supporting Information (Fig. S15**)**. To further reveal the response time, the transient reflectivity change of the spot center is plotted in Fig. 3p. The inset is the transient reflectivity change versus delay time and position. Considering the background noise, the criterion to judge the beginning of decrease is set as Δ*R*/*R*_0_ larger than 10% (the black dashed line in Fig. 3p). It is shown that the reflectivity decreases at approximately 50 ps after laser excitation, indicating that the response time of Sb is about 50 ps. The spatial reflectivity profile shown in Fig. 3q further confirms the low reflectivity region at different delay time. The Gaussian fitting results are consistent with the energy distribution of pump laser.

**Fig. 4:**
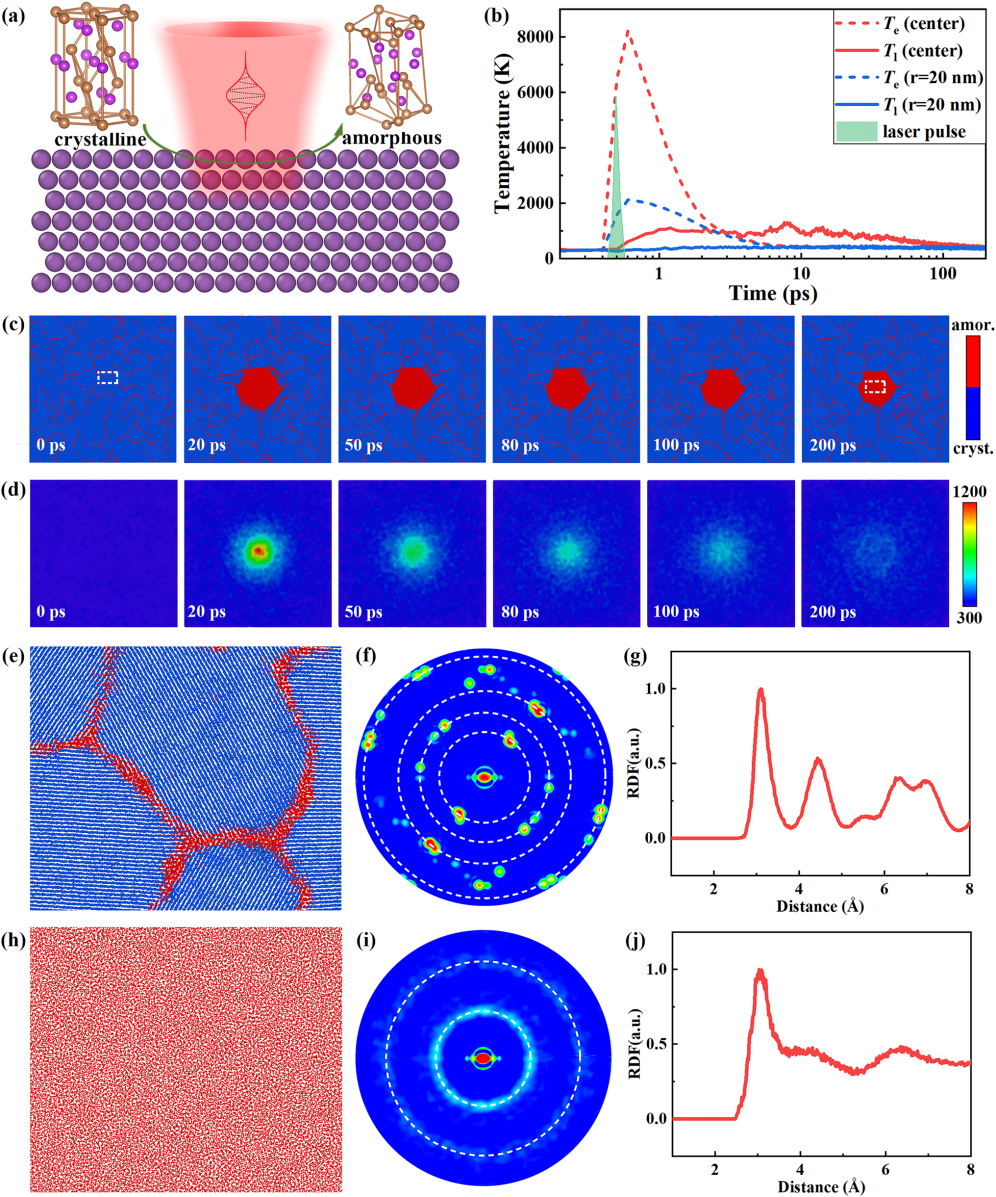
Atomic structure evolution analysis during amorphization. **a** Illustration of structure change during laser-induced amorphization. **b** Temperature evolution of Sb film after laser irradiation. **c** Atomic structure evolution of Sb film after laser irradiation. **d** Temperature distribution of Sb film after laser irradiation. **e** Atomic structure of Sb film at 0 ps (enlarged view of rectangle region in c). **f** Calculated XRD pattern of Sb film at 0 ps. **g** Calculated radius distribution function of Sb film at 0 ps. **h** Atomic structure of Sb film at 200 ps (enlarged view of rectangle region in c). **i** Calculated XRD pattern of Sb film at 200 ps. **j** Calculated radius distribution function of Sb film at 200 ps

To further explore the atomic structure evolution during laser-induced amorphization, molecular dynamics simulations were performed. The details of simulation setup are given in Supporting Information. A single-pulse irradiation is considered in the simulation to evaluate the laser-induced amorphization process, as the illustration in Fig. 4a. The initial dimensions of simulation systems are 5 × 100 × 100 nm^3^, which consists of ≈1.8 million Sb atoms. The electron and lattice temperature are plotted in Fig. 4b. The electron temperature increased to 8000 K within 200 fs, and then, the energy transferred from hot electron to lattice via electron–phonon coupling. The lattice temperature increased to 1200 K in 2 ps, and cooled down to 400 K in 200 ps. The cooling rate is larger than 4 × 10^12^ K s^−1^, which provides a strong quenching effect. The rapid cooling rate enabled the formation of amorphous phase. Figure 4c shows the atomic snapshots of the system after laser excitation. The blue and red atoms belong to crystalline phase and amorphous phase, respectively. The atoms type is determined by their centrosymmetry parameter. The initial system shows a polycrystalline structure, as shown in Fig. 4c. The red atoms at 0 ps are the grain boundary. After laser irradiation, the lattice temperature increased above 1200 K, as shown in Fig. 4d. As the lattice temperature is larger than melting point, materials melting occurred and the crystalline atoms changed to disorder atoms. Due to thermal conduction, the lattice temperature of laser irradiated area decreased from 1200 to 400 K within 200 ps. After the quenching process, the melting materials maintained amorphous structure although the system had cooled below the melting point. The atomic structure of the laser irradiated area before and after laser irradiation is extracted and plotted in Fig. 4e, h. As shown in Fig. 4e, the atoms before laser irradiation are in ordered arrangement, and the lattice fringes can be observed. The two-dimensional diffraction pattern of the atomic configuration in Fig. 4e is calculated and presented in Fig. 4f. Clear crystalline diffraction spots are shown in Fig. 4f, and three diffraction patterns with different zone axis can be found, which is agreed with the polycrystalline structure. The radius distribution function in Fig. 4g also confirms the crystalline phase before laser irradiation. After laser irradiation, the lattice fringes disappear and the atoms show a disorder arrangement, as presented in Fig. 4h. The diffraction pattern in Fig. 4i shows a weak diffraction ring, which is the typical diffraction pattern of amorphous phase. The second peak of the radius distribution function is absent in Fig. 4j, which suggests that the long-range order of atoms is absent after laser irradiation. To evaluate the transition time from amorphous to crystalline phase, the amorphous structure was kept at 500 K for 5 ns (according to the simulation results in Fig. 2a). The simulation results are provided in Supporting Information (Fig. S16). It is suggested that the transition time from amorphous to crystalline phase is about 3.4 ns.

### Integrated Photonic Chips for Convolutional Neural Networks

**Fig. 5:**
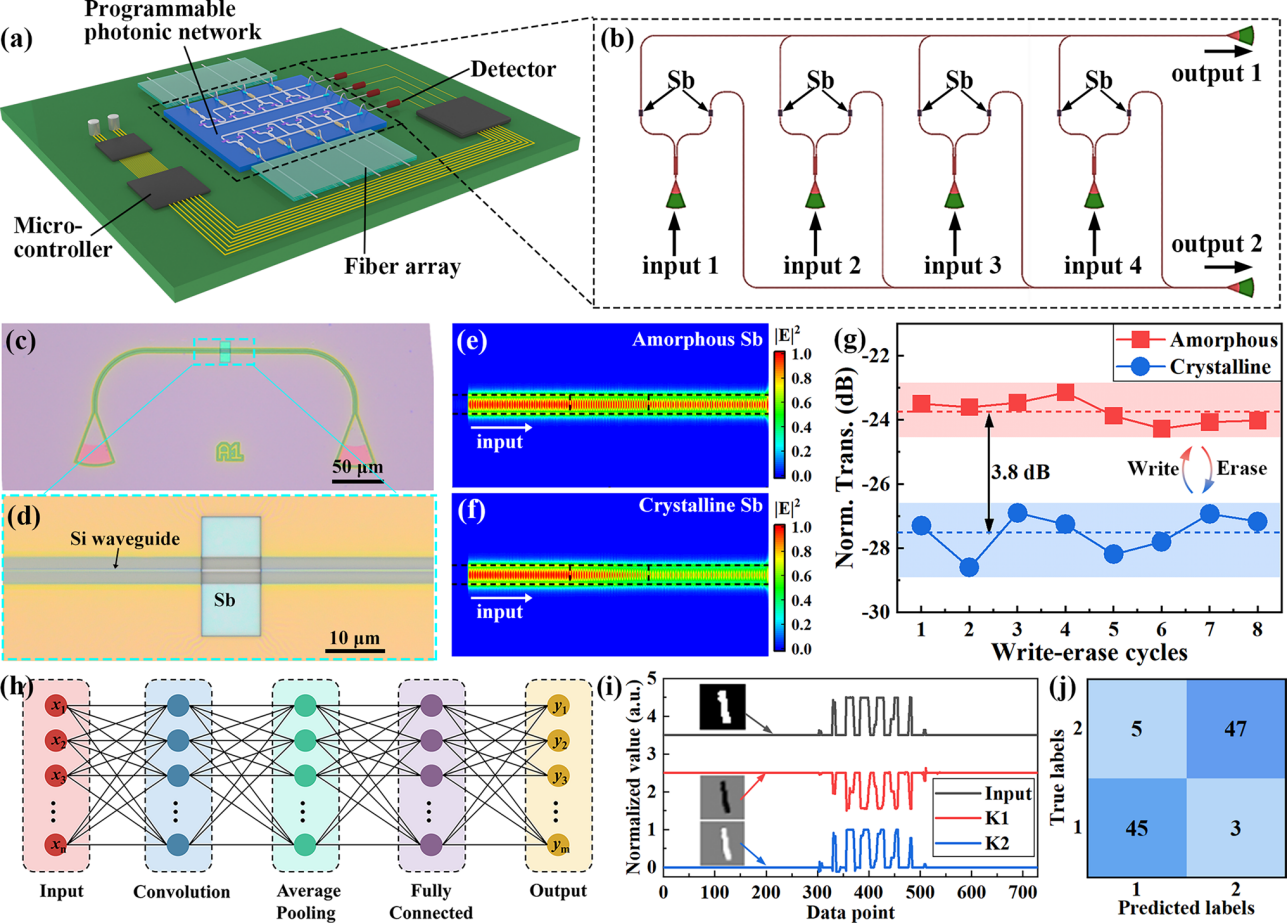
Integrated photonic convolutional network and image recognition. **a** Schematic of photonic network with Sb thin film. **b** Structure of photonic convolutional network consisting of four input channels and two output channels. **c, d** Optical image of the fabricated waveguide with the Sb film deposited. **e, f** Simulated E-field distribution in Si waveguide with crystalline and amorphous Sb film, respectively. **g** Measured transmission of Si waveguide with crystalline (blue) and amorphous (red) Sb film. **h** Architecture and working procedure of neural network algorithms. **i** Input and output signals of the photonic convolutional network. **j** Confused matrix of the designed system

Programmable waveguides are essential components of integrated photonic chips. The large optical contrast between crystalline and amorphous Sb film makes it suitable for programmable optical modulation in integrated photonic chips. As illustrated in Fig. 5a, a photonic convolutional neural network is proposed, which consists of logic control circuit and a 2 × 2 photonic convolutional network. The structure of photonic network is plotted in Fig. 5b, which consists of four input channels and two output channels. The intensity of the input light is modulated by controlling the phase state of Sb film on waveguide. The modulated light then combines into two output channels, and the light intensity is detected by two photodetectors. Figure 5c, d shows the optical images of the fabricated waveguide. To reduce the attenuation of input light, a Sb film with thickness of 5 nm is deposited on the waveguide, and a layer of SiO_2_ with thickness of 5 nm is deposited as protected layer. The width and height of waveguide are 450 and 220 nm, and the dimensions of waveguide and thickness of Sb film are confirmed by atomic force microscope, as shown in Supporting Information (Fig. S17). A 1550-nm CW laser is propagated through the waveguide as signal carrier, while a femtosecond laser is used to switch the phase of Sb film. The light intensity distribution in Si waveguide is calculated by finite element method, as presented in Fig. 5e, f. It is shown that the transmission of waveguide with amorphous Sb film is larger than the waveguide with crystalline Sb film. The change in transmission is due to the difference in extinction coefficient. As the crystalline film has a larger extinction coefficient (*k* = 0.51 for amorphous film and *k* = 2.17 for crystalline film), the crystalline film absorbs more energy when the light evanescently coupled with the Sb film. The simulation results show that the light transmission of Si waveguide can be modulated by switching the phase of Sb film. The measured normalized transmission of waveguides with crystalline and amorphous Sb film is plotted in Fig. 5g. It is demonstrated that the waveguides with amorphous Sb film have a larger transmission than the waveguides with crystalline Sb film. The difference in normalized transmission exceeds 3.8 dB.

Based on the amplitude modulation, a photonic network is designed to perform the convolutional operations, as shown in Fig. 5b. By controlling the phase of Sb film on waveguide, the transmission of weight can be modulated. When the optical signals propagate through the waveguide, the amplitude of signal is modulated and combined at output ports. Based on this photonic network, we have performed a proof-of-concept image recognition task using a convolutional neural network algorithm. The handwritten numbers “1” and “2” from the MNIST database were used to train the model, and the training results are shown in Supporting Information (Fig. S18). The architecture of the model is illustrated in Fig. 5h. The convolutional layer is achieved by the photonic network, and the working principle is shown in Supporting Information (Fig. S19). The input light is split by the 50:50 coupler and propagates through four pairs of waveguides deposited with Sb film. Taking the waveguides of input channel 1 as example, the transmission of waveguide 1 and waveguide 2 was marked as $t_{1}$ and $t_{2}$. By controlling the fraction of crystalline and amorphous phase of Sb film, the transmission was modulated as $w_{11} = t_{1} - t_{2}$, where $w_{11}$ is the element of the kernel matrix. The fraction of crystalline and amorphous phase can be controlled by the laser sweeping area. When the optical signals with intensity *I*_1_ propagate through input channel 1, the light intensity difference between output channel 1 and output channel 2 is $I_{1} w_{11}$. As shown in Fig. S19, by modulating the transmission of the other three input channels to the elements of the kernel matrix, the matrix multiplication is achieved. The 28 × 28 pixels grayscale images are encoded as time series optical signals, and fed into the four input channels, respectively. The output signals are measured by photodetectors and converted to convolution results. The input and output signals of the two photonic kernel K1 and K2 are plotted in Fig. 5i, and the corresponding images decoded from the signals are shown in the insets. The results of handwritten number “2” are plotted in Supporting Information (Fig. S20). The photonic kernels have extracted the images features successfully. The output signals of photonic kernels are added bias and applied the nonlinear ReLU function, then sent to an average pooling layer. Finally, a fully connected layer is used to identify the labels of input images. The confusion matrix for 100 test images (50 number “1” and 50 number “2”) is shown in Fig. 5j, suggesting a 92% recognition accuracy of the systems. These results have demonstrated the potential of Sb thin film in building photonic convolutional network for machine learning applications. As a proof of concept, a small system was built in this work. By increasing the number of phase control units, the system can be expanded to a large system for complex computation task, which will be explored in the future work.

## Conclusions

In summary, a photonic waveguide was fabricated to build integrated photonic networks for machine learning tasks. The laser-induced heating and quenching process provides a cooling rate up to 4 × 10^12^ K s^−1^, resulting in the formation of amorphous Sb film. The phase change timescale of amorphization is estimated to be 50 ps by combining theoretical calculations and experimental measurements. By switching the Sb film between crystalline and amorphous phases, a contrast of 0.65 in refractive index and 1.66 in extinction coefficient at telecommunication wavelength is realized. The large optical contrast between amorphous and crystalline phases enables the modulation of photonic waveguide, and a difference of 3.8 dB is achieved in optical transmission. Photonic convolutional neural networks are constructed using the integrated photonic chips, which is applied for images recognition tasks. This work opens a pathway for programming photonic devices by ultrafast laser, and provides a promising paradigm to create photonic devices for practical photonic computing applications.

## Supplementary Information

Below is the link to the electronic supplementary material.

Supplementary file1 (DOCX 18876 KB)
