# Supplementary material for: Photonic Chip Based on Ultrafast Laser-Induced Reversible Phase Change for Convolutional Neural Network
Source: Nanomicro Lett. 2025 Mar 11;17:179. doi: 10.1007/s40820-025-01693-5 (PMC11896963; doi:10.1007/s40820-025-01693-5)
Supplement: Supplementary file 1 — Supplementary file1 (DOCX 18876 KB) [file 40820_2025_1693_MOESM1_ESM.docx]

Supporting Information for

**Photonic Chip based on Ultrafast Laser Induced Reversible Phase Change for Convolutional Neural Network**

Jiawang Xie^1, 2^, Jianfeng Yan^1, 2, *^, Haoze Han^1, 2^, Yuzhi Zhao^1, 2^, Ma Luo^1, 2^, Jiaqun Li^1, 2^, Heng Guo^1, 2^, and Ming Qiao^1, 2^

^1^ State Key Laboratory of Tribology in Advanced Equipment, Tsinghua University, Beijing 100084, P. R. China

^2^ Department of Mechanical Engineering, Tsinghua University, Beijing 100084, P. R. China

*Corresponding author. E-mail: [yanjianfeng@tsinghua.edu.cn](mailto:yanjianfeng@tsinghua.edu.cn) (Jianfeng Yan)

**S1 TTM-MD simulation**

To study the phase change process of Sb film under ultrafast laser irradiation, a two-temperature model coupled with molecular dynamics (TTM-MD) method was established. Two temperature model (TTM) is widely used to describe the interaction between ultrafast laser and metal. When femtosecond laser irradiates on metal, the free electrons in metal absorb the photon through photon-electron interactions within the pulse duration. It takes a few femtoseconds for electrons to re-establish the Fermi distribution. The energy then transports to the lattice through electron-phonon coupling. As the atoms mass is larger than the electron mass, it takes longer to transport energy from free electrons to the lattice. Typically, it takes a few tens picoseconds for the electrons and the lattice to reach thermal equilibrium. To describe this nonequilibrium process, two temperature model was proposed by Anisimov. In two temperature model, two heat conduction equations are used to calculate the electron and lattice temperature evolution, respectively. They are formulated as:

$C_{e}\left(T_{e}\right)\frac{\partial T_{e}}{\partial t}=\nabla[\kappa_{e}(T_{e})\nabla T_{e}]-G\left(T_{e}-T_{l}\right)+S\left(x,y,z,t\right),$ (S1)

$C_{l}\left(T_{l}\right)\frac{\partial T_{l}}{\partial t}=G\left(T_{e}-T_{l}\right)$, (S2)

where $C_{e}$, $C_{l}$ represents the electron heat capacity, and the lattice heat capacity, respectively. $T_{e}$ is electron temperature, $T_{l}$ is lattice temperature, $\kappa_{e}$is the electron heat conductivity, and *G* is the electron-phonon coupling factor. $S\left(x,y,z,t\right)$ is the laser source term, which is given as:

$S\left(x,y,z,t\right)\text{=}\frac{0.94F}{2\tau_{p}\delta_{p}}\left(1-R\right)\exp\left(-\left(4\text{ln2}\right)\left(\frac{t-t_{0}}{\tau_{p}}\right)^{2}-\frac{\left|x-x_{0}\right|}{\delta_{p}}\right)\exp\left(\frac{\left(y-y_{0}\right)^{2}+\left(z-z_{0}\right)^{2}}{r_{0}^{2}}\right),$ (S3)

where, *F* is the laser fluence, $\tau_{p}$ is the pulse duration, *R* is the reflectivity, $\delta_{p}$ is the optical penetration depth, $t_{0}$ is the moment of initial pulse irradiated on the Sb film. The laser is propagated along *x* axis, and $x_{0}$ is the position of irradiated surface, and $\left(y_{0},z_{0}\right)$ is the spot center.

To solve the equations, the thermal parameters should be calculated first. Generally, the thermal parameters are depended on the electron and lattice temperature. The lattice heat capacity can be calculated by the Dulong-Petit law:

$C_{l}=\frac{3k_{B}}{V_{a}}$, (S4)

where *k_B_* is the Boltzmann constant and *V_a_* is the atomic volume of Sb at specific temperature.

When the electron temperature is less than Fermi temperature, the electron heat capacity can be calculated by the free electron gas model, which shows a linear dependence on electron temperature:

$C_{e}\left(T_{e}\right)=\gamma T_{e},$ (S5)

where *γ* is a constant determined by:

$\gamma=\frac{\pi^{2}n_{e}k_{B}^{2}}{2\varepsilon_{F}},$ (S6)

where is $n_{e}$ free electron density, $\varepsilon_{F}$ is Fermi energy.

During ultrafast laser irradiation, the electron temperature can reach to 10^4^ K, which is closed or above the Fermi temperature, Equation (S5) is not suitable to describe the thermal parameters in this condition. A more general formulation is adopted to calculated the electron heat capacity:

$C_{e}\left(T_{e}\right)=\int_{-\infty}^{\infty} \frac{\partial f\left(\varepsilon,\mu,T_{e}\right)}{\partial T_{e}}g\left(\varepsilon\right)\varepsilon d\varepsilon$ (S7)

where *ε* is the energy state of electron, *g*(*ε*) is the density of state, *μ* is the chemical potential, and *f* is the Fermi distribution function, which is formulated as:

$f\left(\varepsilon,\mu,T_{e}\right)=\frac{1}{e^{\frac{\varepsilon-\mu}{k_{B}T_{e}}}+1}$ (S8)

As the number of valence electrons is conserved, the chemical potential *μ* can be calculated by solving the following equation:

$N=\int_{-\infty}^{\infty} f\left(\varepsilon,\mu\left(T_{e}\right),T_{e}\right)g\left(\varepsilon\right)d\varepsilon$ (S9)

The electron-phonon coupling factor is calculated as:

(S10)

where is the reduced Planck constant, $\lambda$ is the electron-phonon coupling constant (0.17 for Sb), $<\omega^{2}>$ is the second moment of phonon spectrum, which can be estimated by the Deby temperature $\theta_{D}$:

$<\omega^{2}>\approx\frac{\theta_{D}^{2}}{2}$ (S11)

According to Equations (7) and (10), it requires the knowledge of the density of state (DOS) to calculated the electron temperature dependence of *C_e_* and *G*. The DOS of Sb can be obtained using density functional theory, as implemented in the ABINIT code. **Figure S1** has shown the DOS, *μ*, *C_e_*, and *G* of Sb.

TTM has been successfully applied to calculate the temperature evolution of metal irradiated with femtosecond laser. However, it cannot investigate the phase change process of materials. To study the change of structure, a two-temperature model coupled with molecular dynamics (TTM-MD) method is proposed. The coupling TTM-MD method calculates the electron temperature through Equation (S1), while the lattice temperature is obtained from molecular dynamics simulation. Equation (S1) is solved by finite difference method (FDM) simultaneously with the MD integration. To simulate the coupling process of electron and lattice, a coupling term is added to the atoms motion equation:

$m_{i}\frac{d^{2}\mathbf{r}_{i}}{dt^{2}}=\mathbf{F}_{i}+\xi m_{i}\mathbf{v}_{i}^{T},$ (S16)

where $m_{i}$, $\mathbf{r}_{i}$ and $\mathbf{v}_{i}^{T}$ are the mass, position, thermal velocity of atom *i*, respectively. $\mathbf{F}_{i}$ is the force acting on atom *i* due to the interatomic interaction. $\xi m_{i}\mathbf{v}_{i}^{T}$ is an additional force term coming from the electron-phonon coupling. The coupling coefficient is given as:

$\xi=\frac{G\sum_{k=1}^{n} V_{N}\left(T_{e}^{k}-T_{l}\right)}{n\sum_{i} m_{i}\left(v_{i}^{T}\right)^{2}},$ (S17)

where $V_{N}$ is the volume of each cell used for FDM simulation, and *n* is the total number of FDM cells.

The lattice temperature is calculated from the kinetic energy of atoms:

$KE\text{=}\frac{\dim}{2}N_{a}k_{\text{B}}T_{l},$ (S18)

where *KE* is the kinetic energy of the systems, and dim is the dimension of the simulation, and *N_a_* is the total number of atoms.

A TTM-MD simulation domain with dimension of 5×100×100 nm^3^, which consists of ≈1.8 million Sb atoms, was built to study the laser-induced phase change process of Sb. Free boundary condition was applied in *x* dimension, whereas periodic boundary condition was applied in *y* and *z* dimension. To reduce the computational cost, the radius of laser spot was selected as 10 nm. Time step for MD simulation was 1 fs and the mesh resolution for FDM calculation was 1 nm. The interatomic interaction between Sb atoms was calculated by the embedded atom method (EAM) potential. Before loading laser energy, the system run to equilibrium state under the microcanonical (NVE) ensemble in 1000 ps.

**S2 Simulation of reflection spectra**

The reflection spectra of Sb thin film were calculated using the transfer matrix method. As shown in **Fig. S2**, when the TE polarization light is incident on the film at an angle of *θ*_0_, the electric field at interface *a* and *b* has the equation as following:

$E_{a}=E_{0}+E_{r1}=E_{t1}+E_{i1}$ (S19)

$E_{b}=E_{i2}+E_{r2}=E_{t2}$ (S20)

The magnetic field at interface *a* and *b* has the equation as following:

$H_{a}=H_{0}\text{cos}\theta_{0}-H_{r1}\text{cos}\theta_{0}=H_{t1}\text{cos}\theta_{1}-H_{i1}\text{cos}\theta_{1}$ (S21)

$H_{b}=H_{i2}\text{cos}\theta_{1}-H_{r2}\text{cos}\theta_{1}=H_{t2}\text{cos}\theta_{2}$ (S22)

The electric field and magnetic field have the following relation:

$H_{i}=\sqrt{\frac{\varepsilon_{i}}{\mu_{i}}}E_{i}=\sqrt{\frac{\varepsilon_{0}}{\mu_{0}}}E_{i}n_{i}$ (S23)

where *ε*_0_ and *μ*_0_ is the dielectric constant and permittivity of vacuum, *n_i_* is the refractivity index of the film.

Combing Equations (S20), (S22) and (S23), we can derive the following equation:

$H_{a}=\sqrt{\frac{\varepsilon_{0}}{\mu_{0}}}\left(E_{0}-E_{r1}\right)n_{0}\cos\theta_{0}=\sqrt{\frac{\varepsilon_{0}}{\mu_{0}}}\left(E_{t1}-E_{i1}\right)n_{1}\text{cos}\theta_{1}$ (S24)

$H_{b}=\sqrt{\frac{\varepsilon_{0}}{\mu_{0}}}\left(E_{i2}-E_{r2}\right)n_{1}\cos\theta_{1}=\sqrt{\frac{\varepsilon_{0}}{\mu_{0}}}E_{t2}n_{2}\text{cos}\theta_{2}$ (S25)

The transmitted electric field *E_t_*_1_ at interface field and the incident electric field *E_i_*_2_ at interface b should satisfy:

$E_{i2}=E_{t1}e^{-iK_{z}d}=E_{t1}e^{i\delta_{1}}$ (S26)

$\delta_{1}=-K_{z}d=\frac{-2\pi}{\lambda_{0}}n_{1}d\cos\theta_{1}$ (S27)

*δ*_1_ is the phase difference of the plane wave at interface *a* and *b*, *d* is the thickness of the film.

Similarly, we can derive:

$E_{i1}=E_{r2}e^{-iK_{z}d}=E_{r2}e^{i\delta_{1}}$ (S28)

Consequently,

$E_{b}=E_{i2}+E_{r2}=E_{t1}e^{i\delta_{1}}+E_{i1}e^{-i\delta_{1}}$ (S29)

$H_{b}=\sqrt{\frac{\varepsilon_{0}}{\mu_{0}}}\left(E_{i2}-E_{r2}\right)n_{1}\cos\theta_{1}=\sqrt{\frac{\varepsilon_{0}}{\mu_{0}}}n_{1}\cos\theta_{1}\left(E_{t1}e^{i\delta_{1}}-E_{i1}e^{-i\delta_{1}}\right)$ (S30)

replace $\gamma_{1}=\sqrt{\frac{\varepsilon_{0}}{\mu_{0}}}n_{1}\cos\theta_{1}$ in Equation (S30), we can obtain:

$E_{t1}=\frac{e^{-i\delta_{1}}}{2}\left(E_{b}+\frac{H_{b}}{\gamma_{1}}\right)$ (S31)

$E_{i1}=\frac{e^{i\delta_{1}}}{2}\left(E_{b}-\frac{H_{b}}{\gamma_{1}}\right)$ (S32)

combing Equations (19), (25), (31), and (32), we can obtain:

$E_{a}=E_{b}\cos\delta_{1}-H_{b}\frac{i\sin\delta_{1}}{\gamma_{1}}$ (S33)

$H_{a}=-E_{b}\gamma_{1}i\sin\delta_{1}+H_{b}\cos\delta_{1}$ (S34)

writing in matrix formulation:

$\left[\begin{matrix} E_{a} \\ H_{a} \end{matrix}\right]=\left[\begin{matrix} \cos\delta_{1} & -\frac{i}{\gamma_{1}}\sin\delta_{1} \\ -i\gamma_{1}\sin\delta_{1} & \cos\delta\end{matrix}\right]\left[\begin{matrix} E_{b} \\ H_{b} \end{matrix}\right]=\mathbf{M}_{1}\left[\begin{matrix} E_{b} \\ H_{b} \end{matrix}\right]$ (S35)

**M_1_** is the transfer matrix of the film.

For a multilayer film system, each layer has a transfer matrix **M*_i_***, the transfer matrix of the multilayer film is:

$\mathbf{M}=\mathbf{M}_{\mathbf{1}}\mathbf{M}_{\mathbf{2}}\mathbf{M}_{\mathbf{3}}\ldots\mathbf{M}_{\mathbf{n}}=\left[\begin{matrix} m_{11} & m_{12} \\ m_{21} & m_{22} \end{matrix}\right]$ (S36)

Using the transfer matrix, the transmission coefficient *t* and reflection coefficient *r* can be calculated:

$t=\frac{2\gamma_{0}}{\gamma_{0}m_{11}+\gamma_{0}\gamma_{S}m_{12}+m_{21}+\gamma_{S}m_{22}}$ (S37)

$r=\frac{\gamma_{0}m_{11}+\gamma_{0}\gamma_{s}m_{12}-m_{21}-\gamma_{s}m_{22}}{\gamma_{0}m_{11}+\gamma_{0}\gamma_{s}m_{12}+m_{21}+\gamma_{s}m_{22}}$ (S38)

The transmission *T* and reflection *R* is calculated as:

$T=\left|t\right|^{2}$ (S39)

$R=\left|r\right|^{2}$ (S40)

**S3 Training the convolutional neural network for handwritten number recognition**

The convolutional neural network was built and train with a back-propagation algorithm using the gradient descent method. The network consists of an input layer, a convolutional layer with two channels, an average pooling layer, a fully connected layer, and an output layer. The nonlinear function ReLu was applied to the convolutional results, and sigmoid function was applied to the fully connected layer. 6742 images of the handwritten number “1” and 5958 images of the handwritten number “2” from the MNIST database were used as the training set. The epoch number of the training was 3000. The training results of all matrices and bias are shown in **Fig. S3**.

The convolution operation was performed by the designed photonic circuits. The kernel matrices were stored in the laser programmed Sb film. By controlling the amorphization ratio of Sb film, the contrast of light intensity of each brunch can be tuned, which was set to the value of each element in kernel matrix. The output light signals were detected by the photodetectors and converted to electrical signals. The post-processing operation, including adding bias, applying nonlinear function, pooling, and fully connected was performed on the output electrical signals through regular electronic computing method. The finally output was a 2×1 vector, with the first element gave the score for the label “1” while the second element gave the score for the label “2”. 50 images of number “1” and 50 images of number “2” were used to test the systems, and 92 out of 100 images was identified correctly.

**S4 Supplementary Figures**


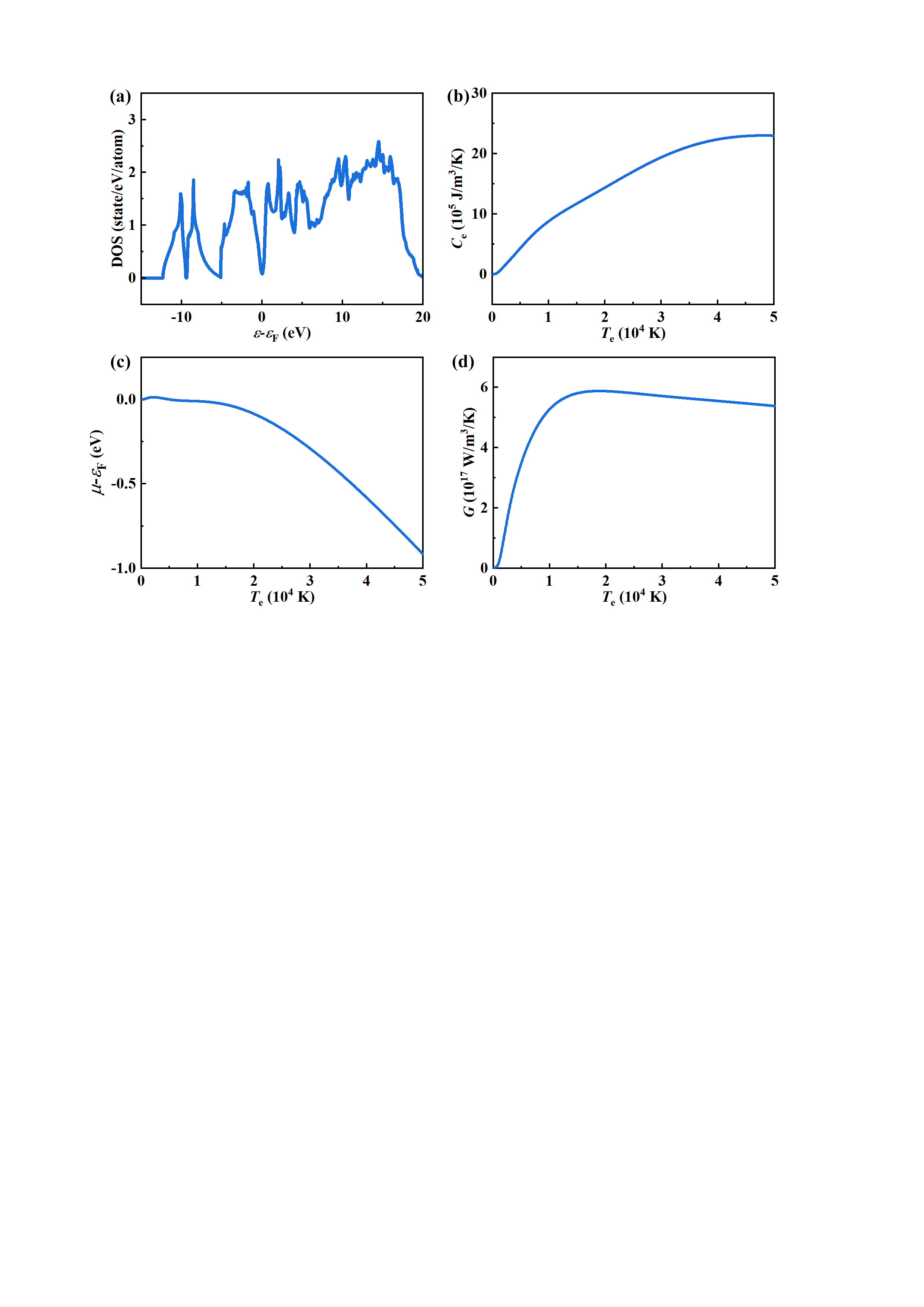


**Fig. S1** (**a**) The electron DOS of Sb obtained by ab initio calculation. (**b**) The electron heat capacity of Sb dependent on electron temperature. (**c**) The chemical potential of Sb dependent on electron temperature. (**d**) The electron-phonon coupling factor of Sb dependent on electron temperature


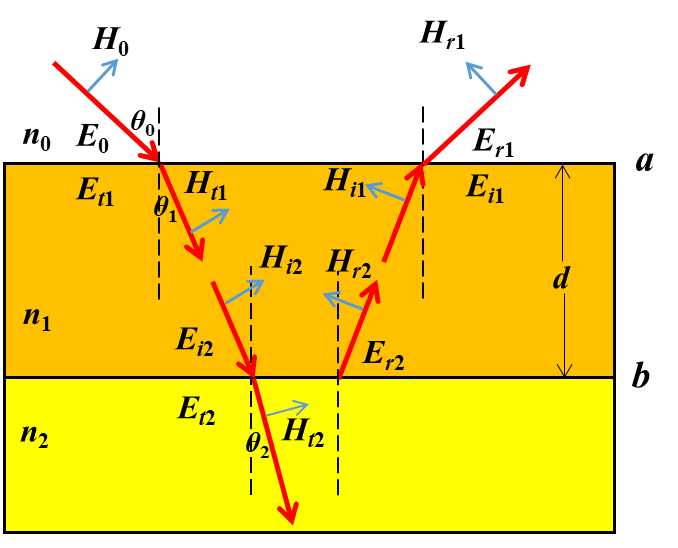


**Fig. S2** Schematic of single layer thin film reflection
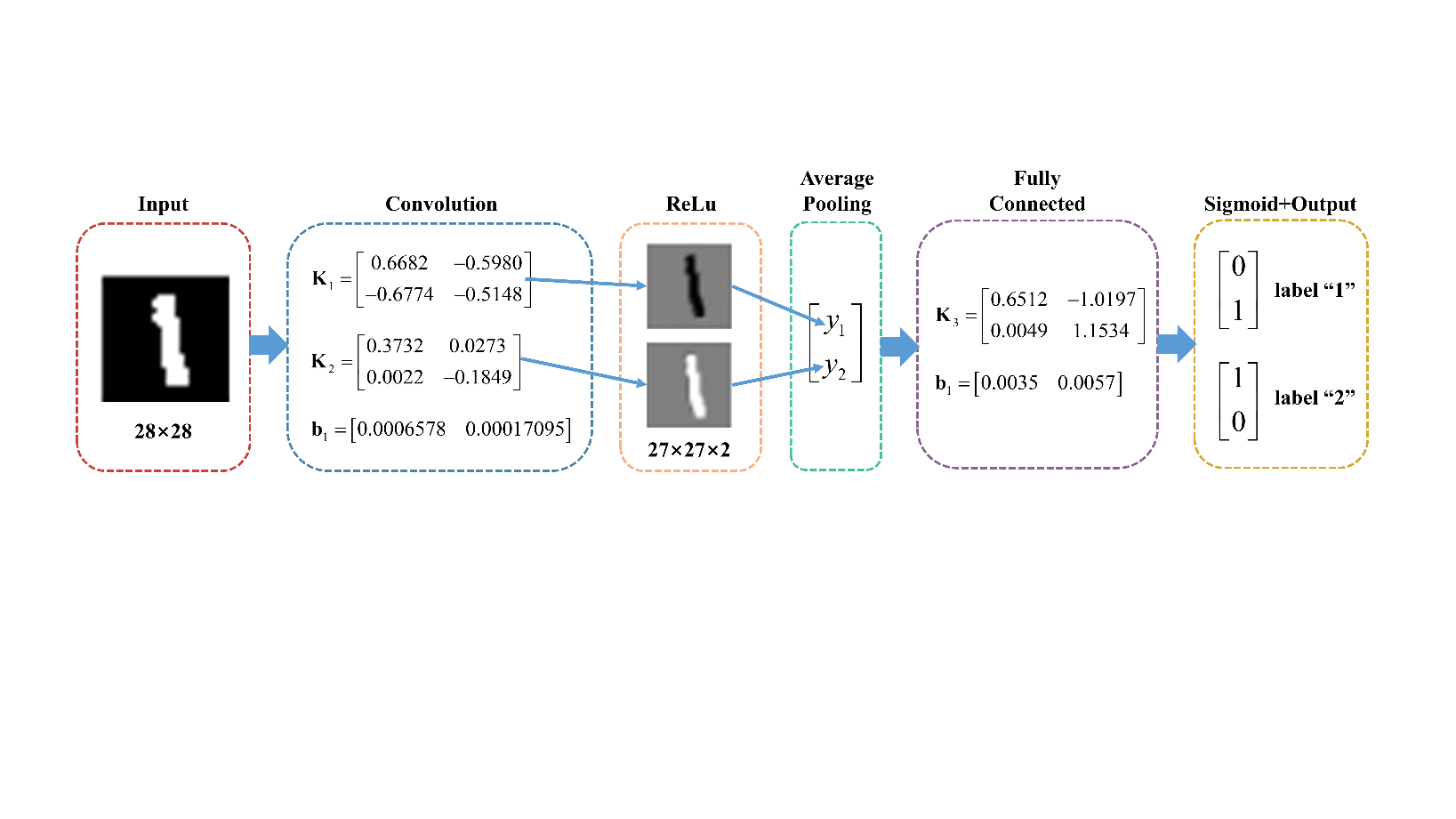


**Fig. S3** Schematic of the convolutional neural network configuration with kernel matrices and bias after training


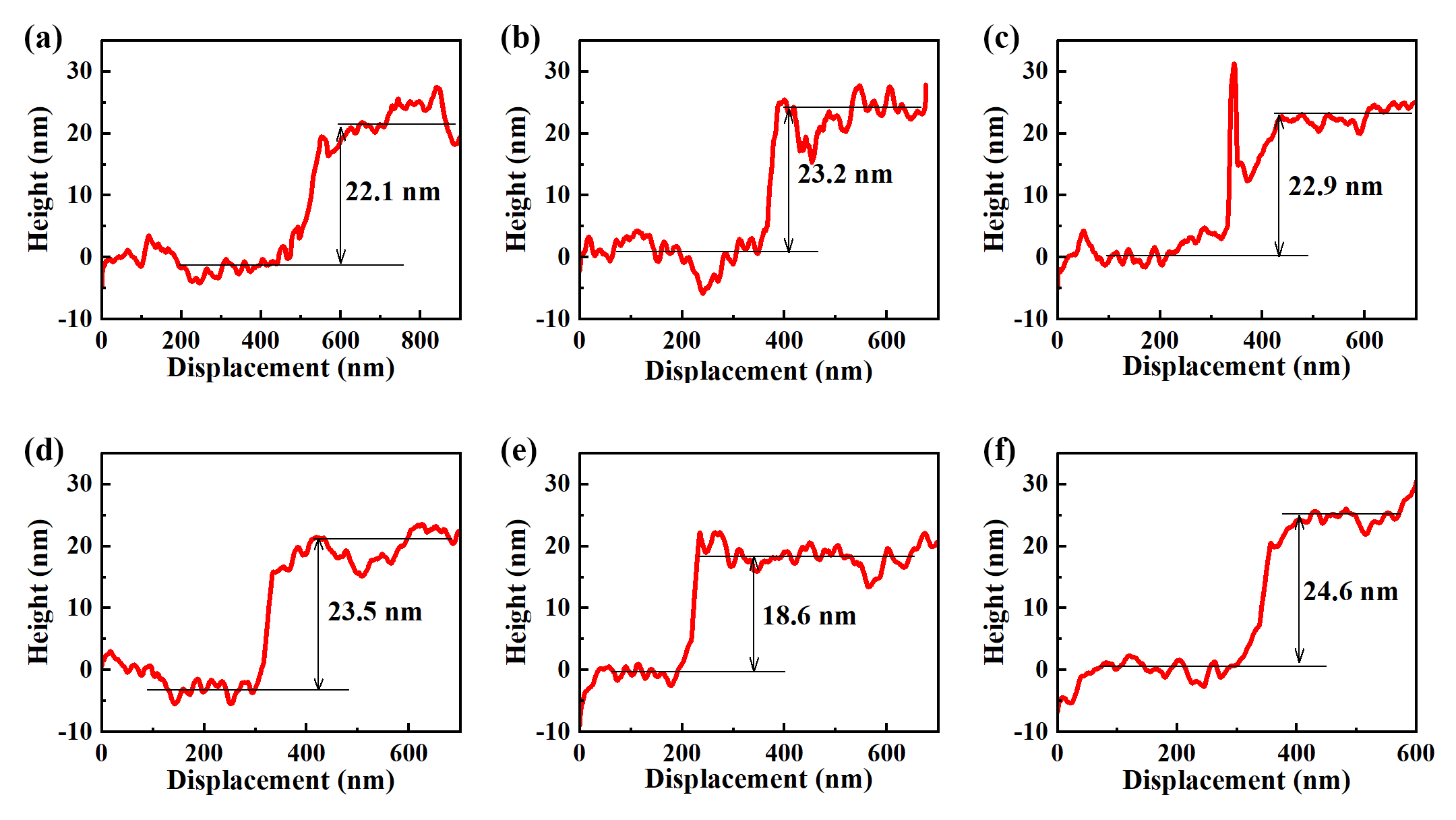


**Fig. S4** Thickness of as-prepared Sb film. (**a**) sample 1; (**b**) sample 2; (**c**) sample 3; (**d**) sample 4; (**e**) sample 5; (**f**) sample 6


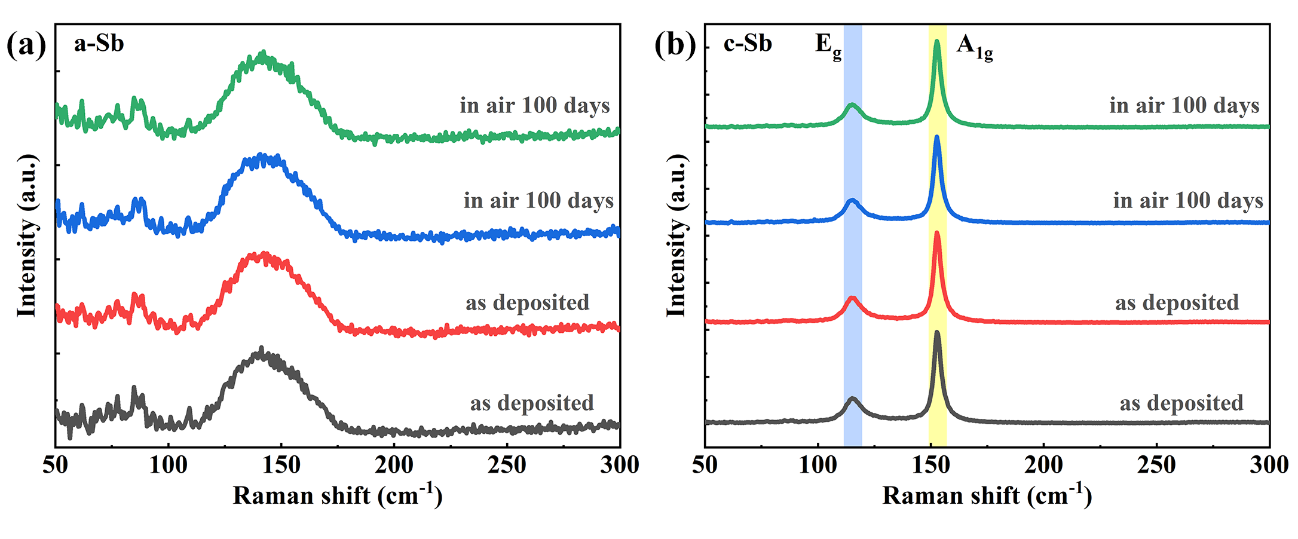


**Fig. S5** Raman spectra of Sb thin film. (**a**) The amorphous film as-deposited and exposured in air for 100 days. (**b**) The crystalline film as-deposited and exposured in air for 100 days. The spectra indicated the Sb film is stable in atmosphere


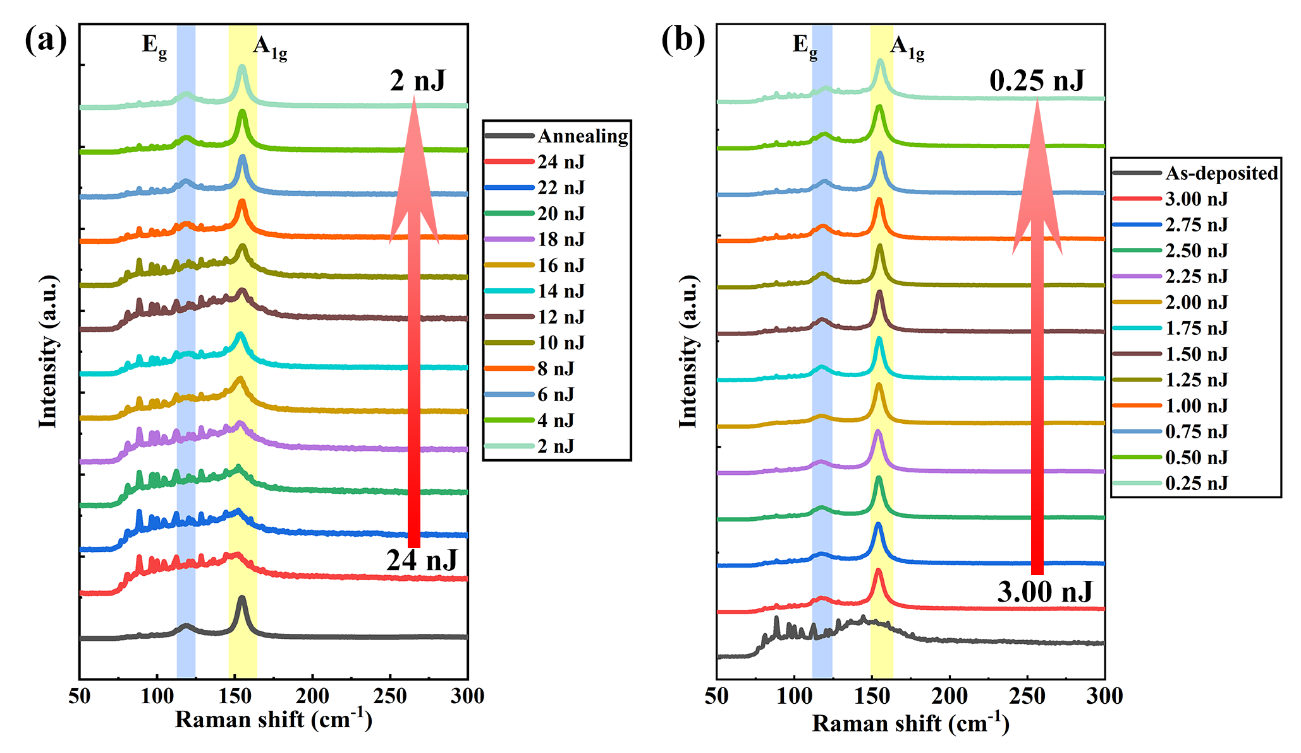


**Fig. S6** Raman sepctra of Sb thin film irradiated by femtosecond laser with various power. (**a**) The spectra of crystalline film irradiated by femtosecond laser from 2 to 24 nJ. The crystalline film changed to amorphous phase when laser power was larger than 10 nJ. (**b**) The spectra of amorphous film irradiated by 80MHz femtosecond laser from 0.25 to 3.00 nJ


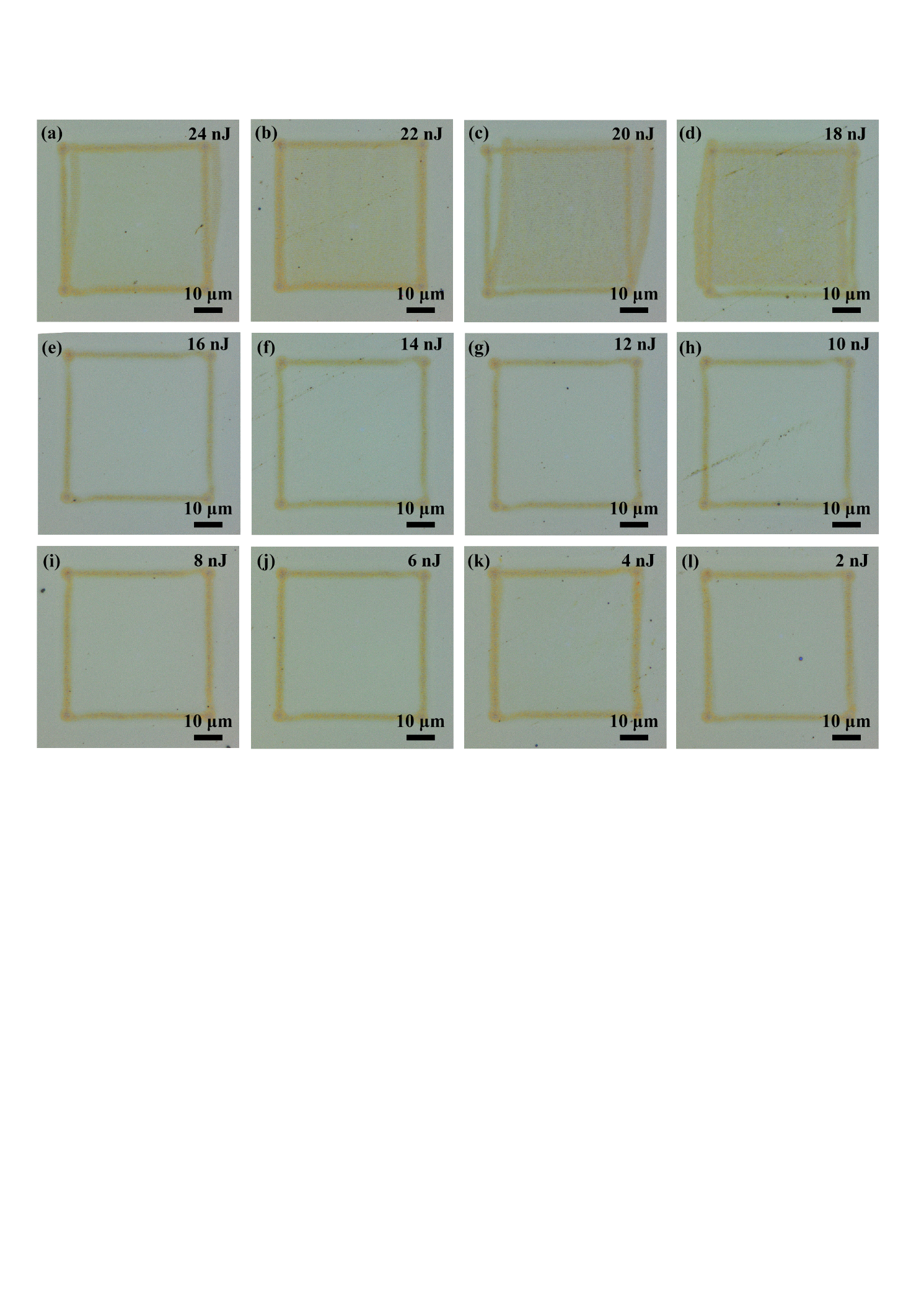


**Fig. S7** Optical images of crystalline Sb film irradiated by femtosecond laser from 2 nJ to 24 nJ. (**a**)-(**l**) is 24 nJ, 22 nJ, 20 nJ, 18 nJ, 16 nJ, 14 nJ, 12 nJ, 10 nJ, 8 nJ, 6 nJ, 4 nJ, 2 nJ, respectively. The optical images indicates the crystalline film is damaged when laser power larger than 16 nJ.

.
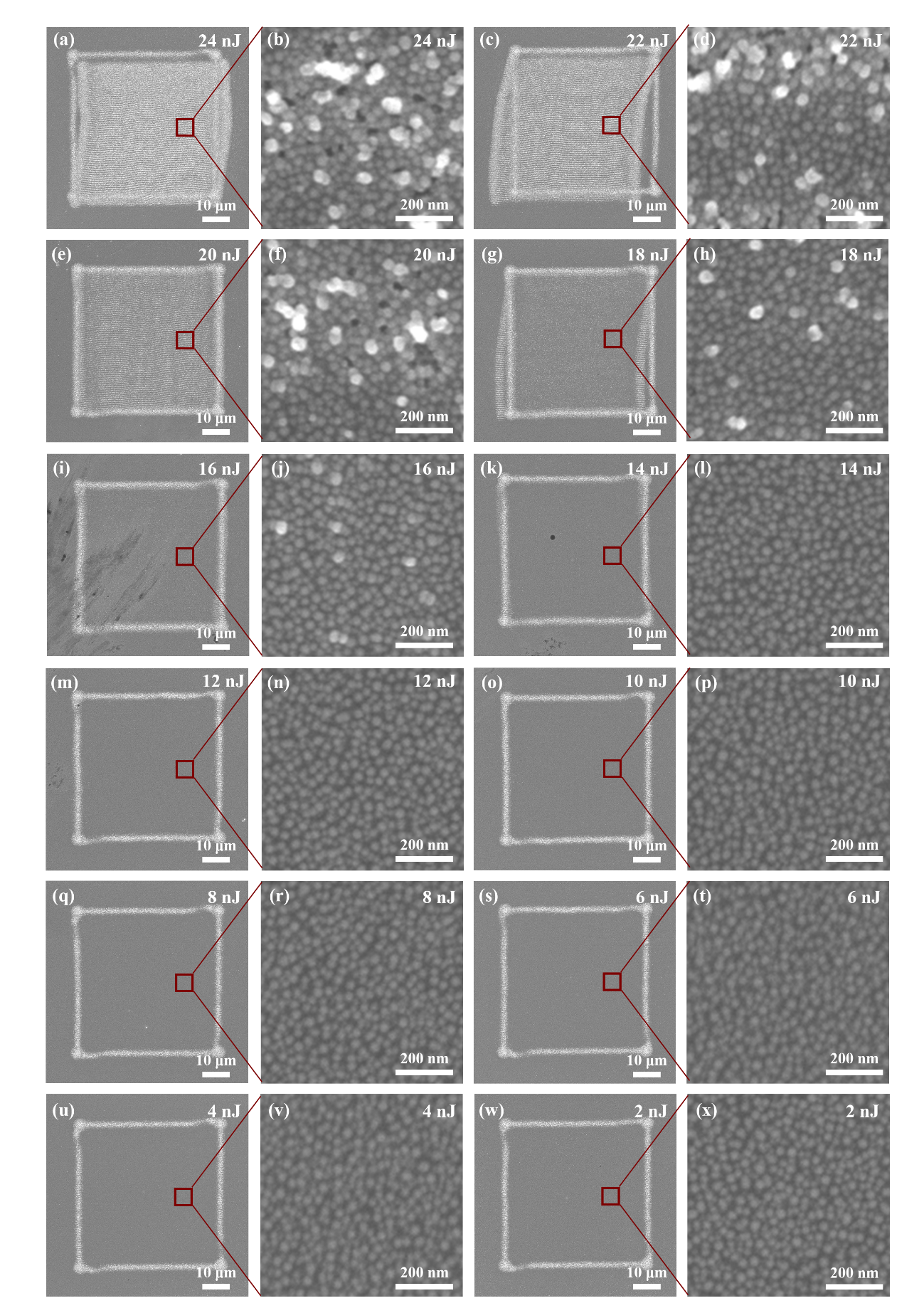


**Fig. S8** SEM images of crystalline Sb film irradiated by femtosecond laser from 2 to 24 nJ. The SEM images indicates the Sb film is damaged when laser power larger than 16 nJ.


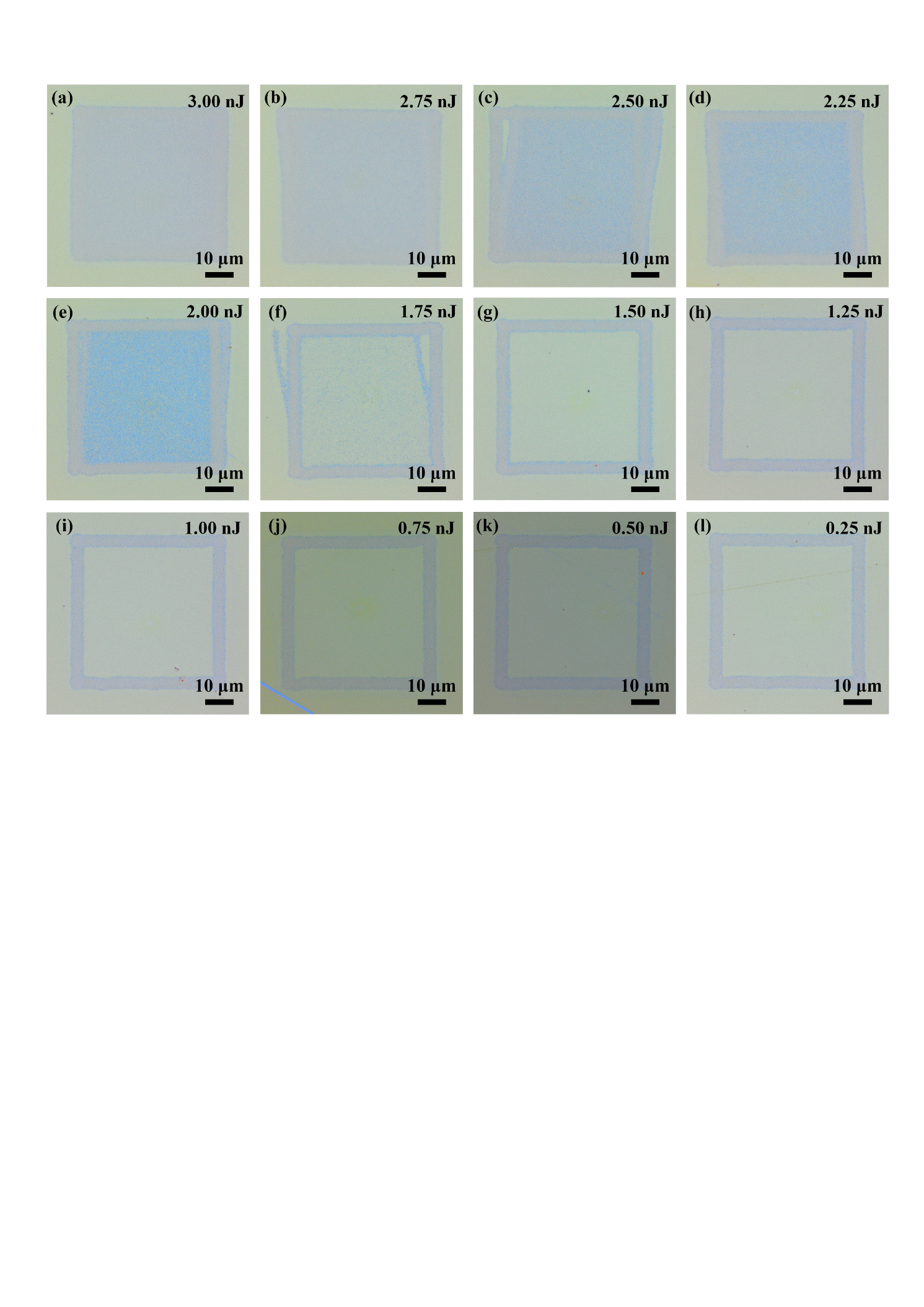


**Fig. S9** Optical images of amorphous Sb film irradiated by 80MHz femtosecond laser from 0.25 nJ to 3.00 nJ. (**a**)-(**l**) is 3.00 nJ, 2.75 nJ, 2.50 nJ, 2.25 nJ, 2.00 nJ, 1.75 nJ, 1.50 nJ, 1.25 nJ, 1.00 nJ, 0.75 nJ, 0.50 nJ, 0.25 nJ, respectively. The optical images imdicates the amorphous film is damaged when laser power larger than 2.00 nJ.


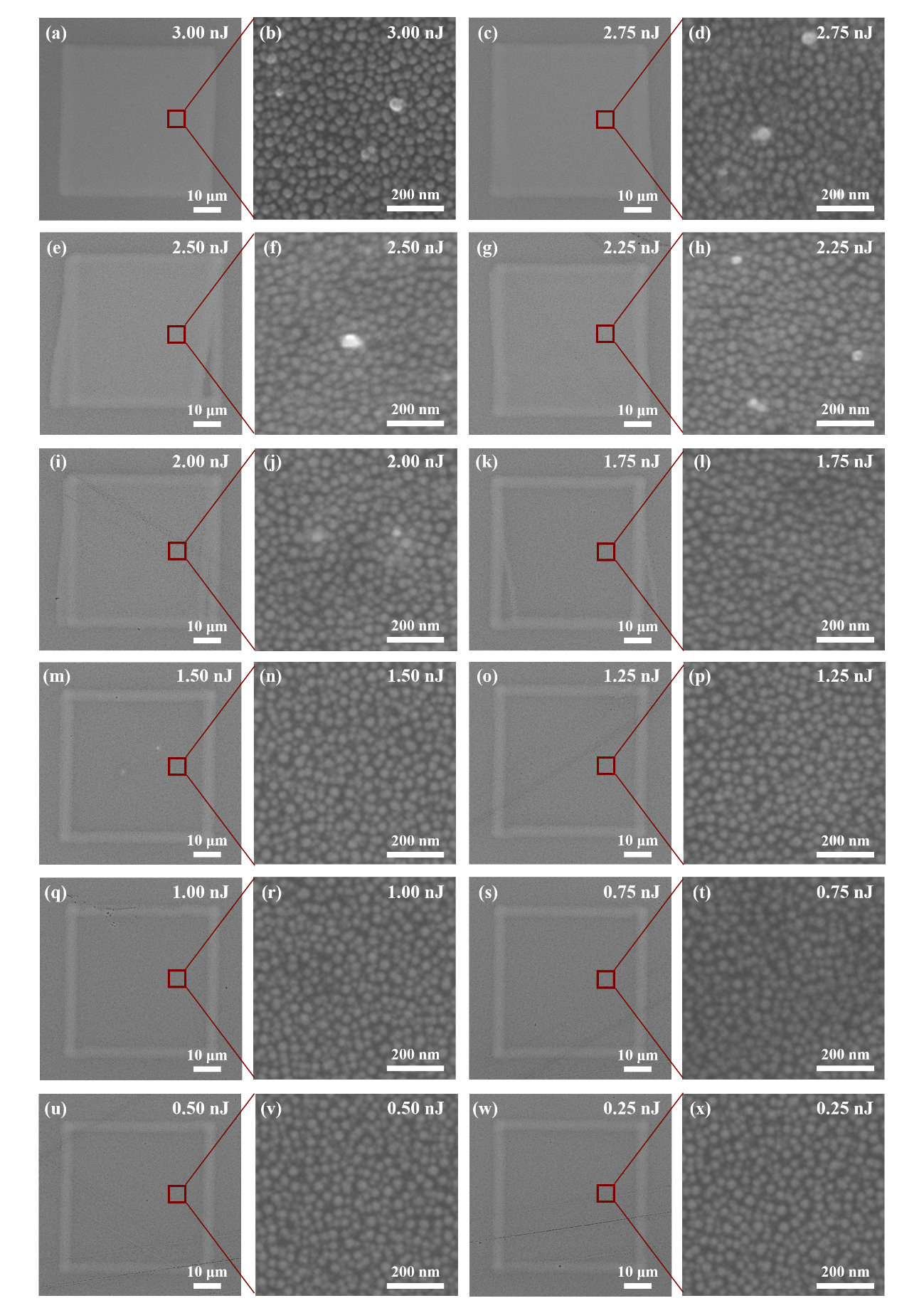


**Fig. S10** SEM images of amorphous Sb film irradiated by 80MHz femtosecond laser from 0.25 nJ to 3.00 nJ. The SEM images indicates the Sb film is damaged when laser power larger than 2.00 nJ.


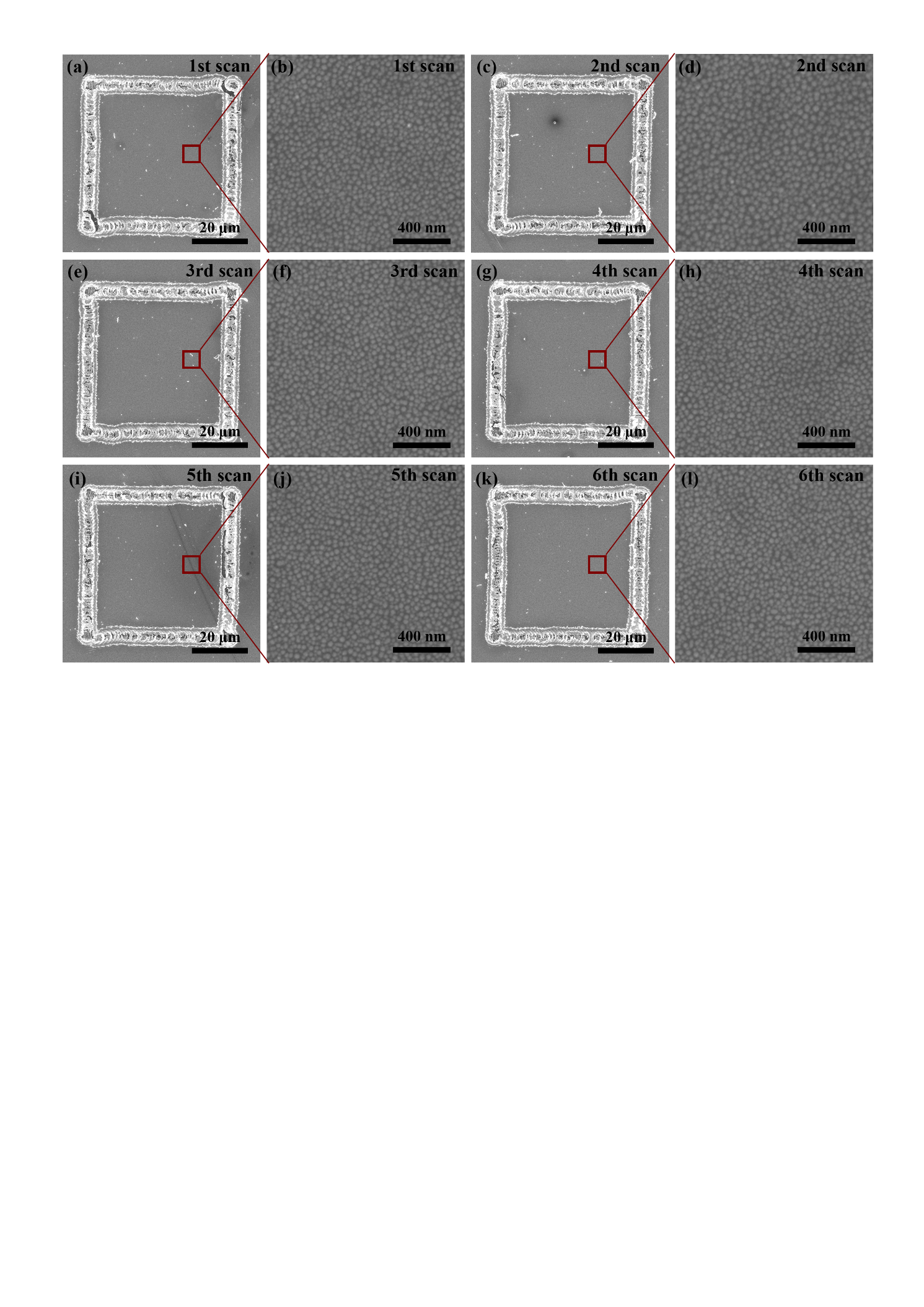


**Fig. S11** SEM images of Sb film irradiated by femtosecond laser with power of 12 nJ and 1.25 nJ for 3 cycles. (**a, b**) After first scan with 12 nJ. (**c, d**) After second scan with 1.25 nJ. (**e, f**) After third scan with 12 nJ. (**g, h**) After fourth scan with 1.25 nJ. (**i, j**) After fifth scan with 12 nJ. (**k, l**) After sixth scan with 1.25 nJ


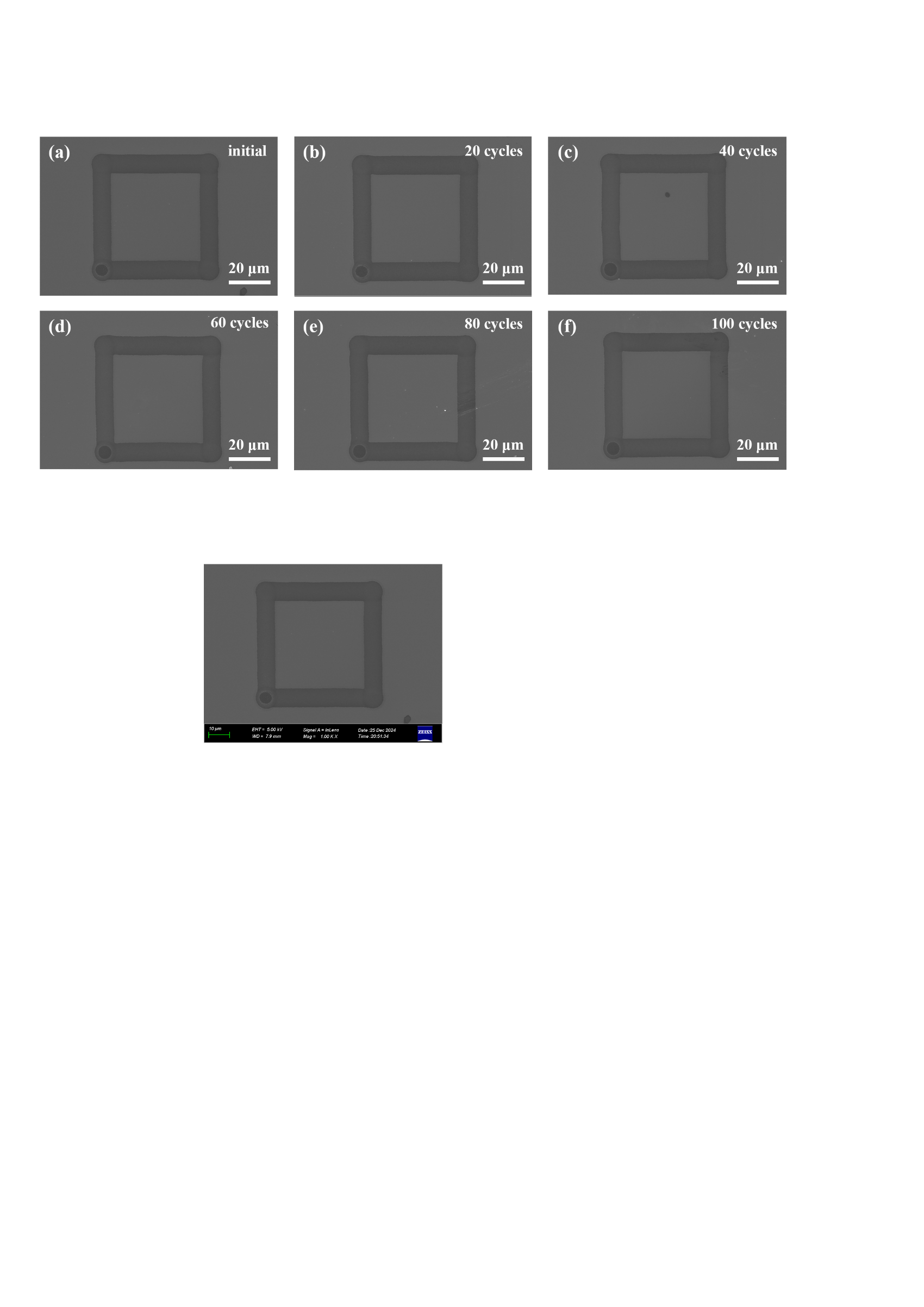


**Fig. S12** SEM images of Sb film irradiated by femtosecond laser with power of 12 nJ and 1.25 nJ for 100 cycles. (**a**) initial film; (**b**) film after 20 switching cycles; (**c**) film after 40 switching cycles; (**d**) film after 60 switching cycles; (**e**) film after 80 switching cycles; (**f**) film after 100 switching cycles


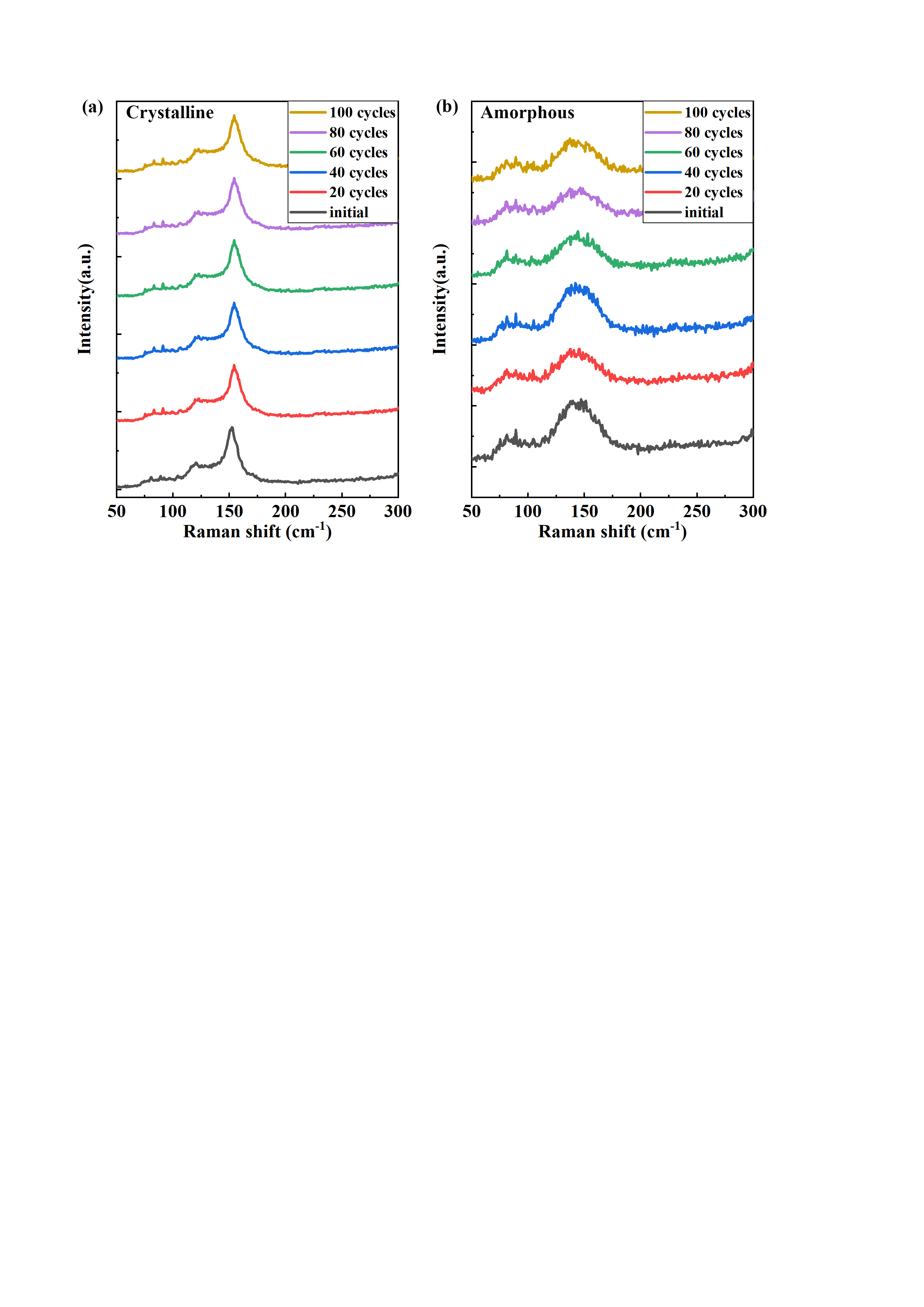


**Fig. S13** Raman spectra of crystalline and amorphous Sb film during 100 switching cycles. (**a**) crystalline film; (**b**) amorphous film


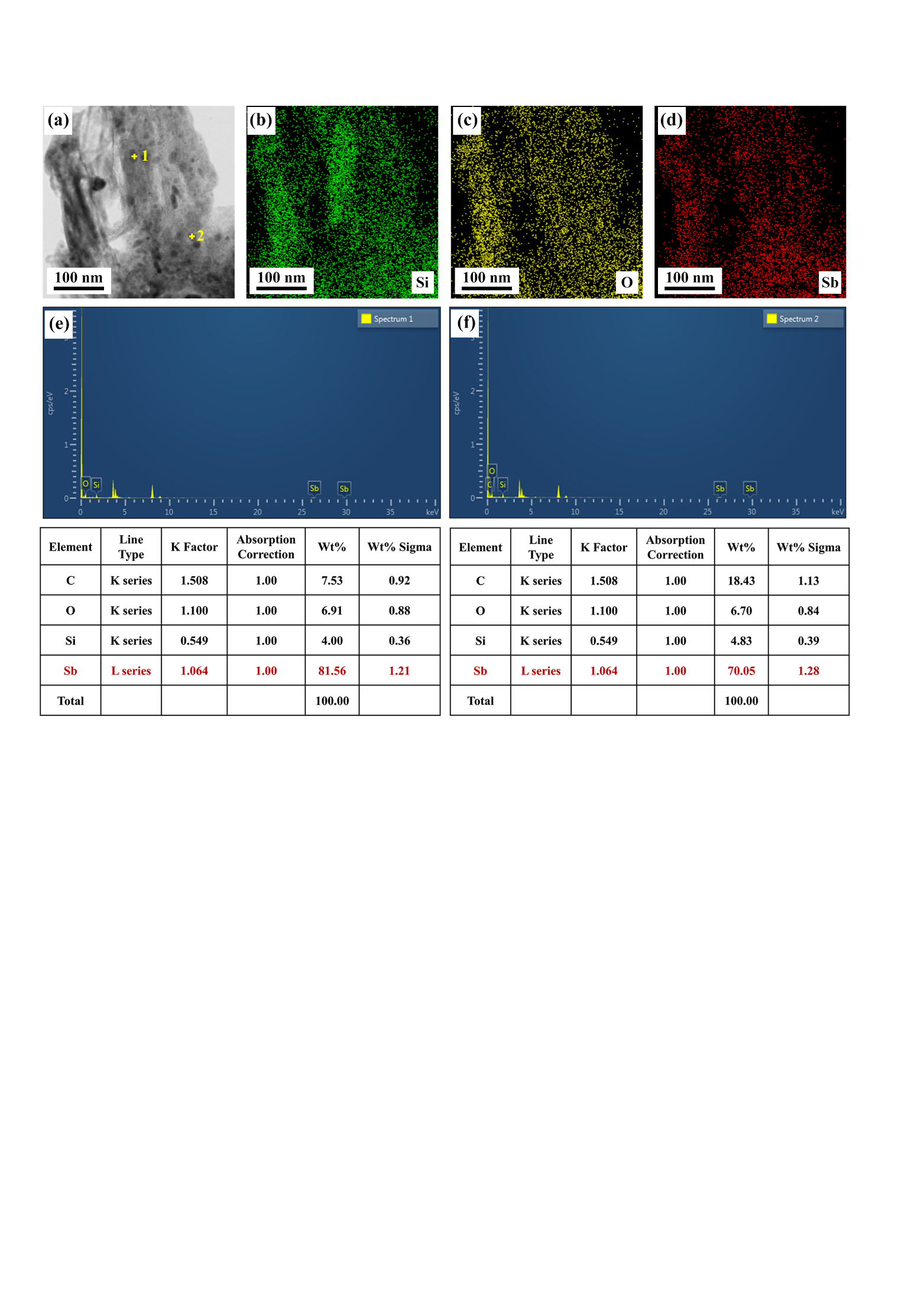


**Fig. S14** EDS characterization of Sb film. (**a**) Electron image. (**b-d**) Elemental mapping of Si, O, and Sb, respectively. (**e, f**) EDS spectra of the position marked in a. The tables show the weight percentage of different elements. The Si and O elements belong to the silica protected layer.


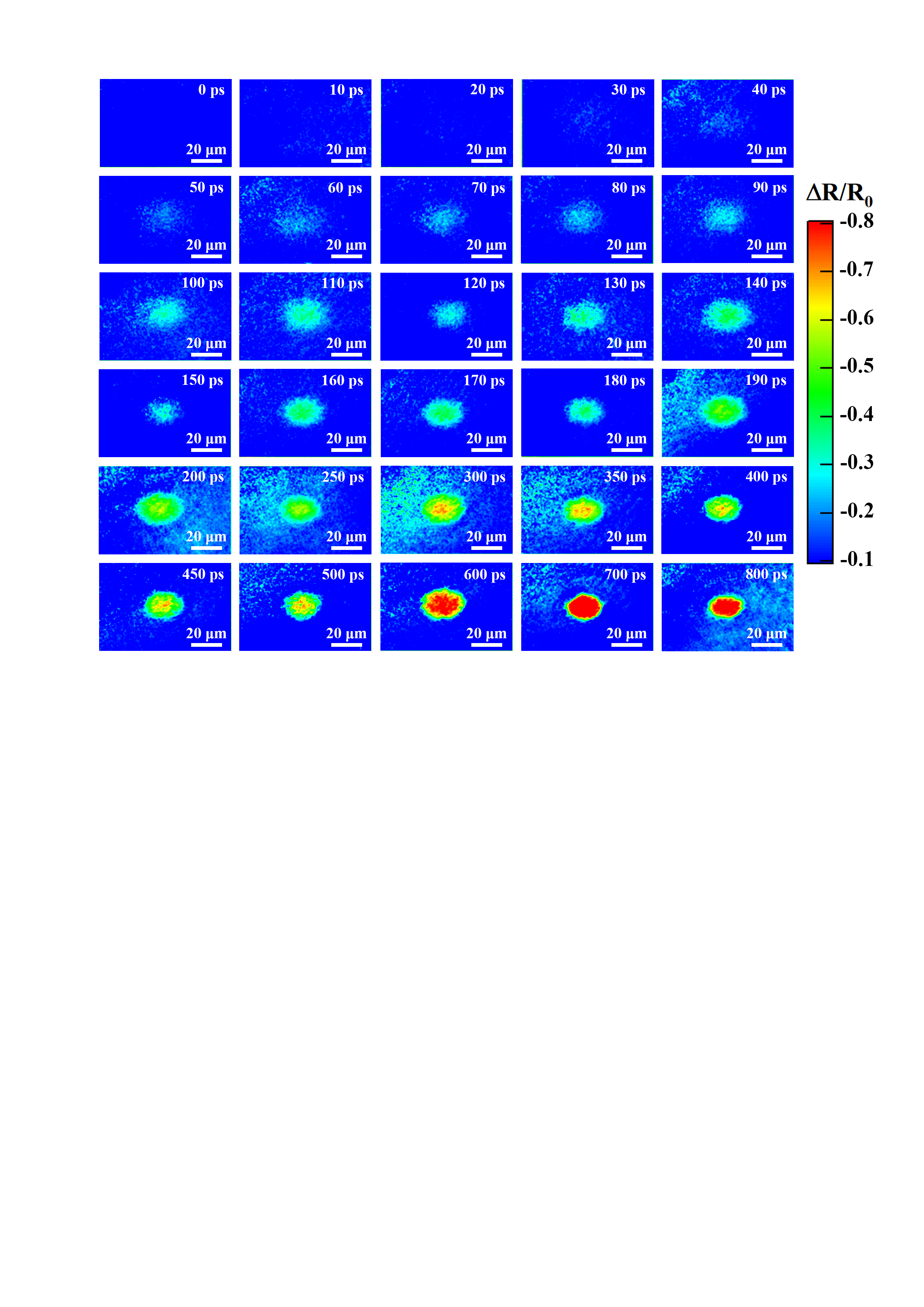


**Fig. S15** Transient reflectivity of Sb film at 0 to 800 ps after laser irradiation


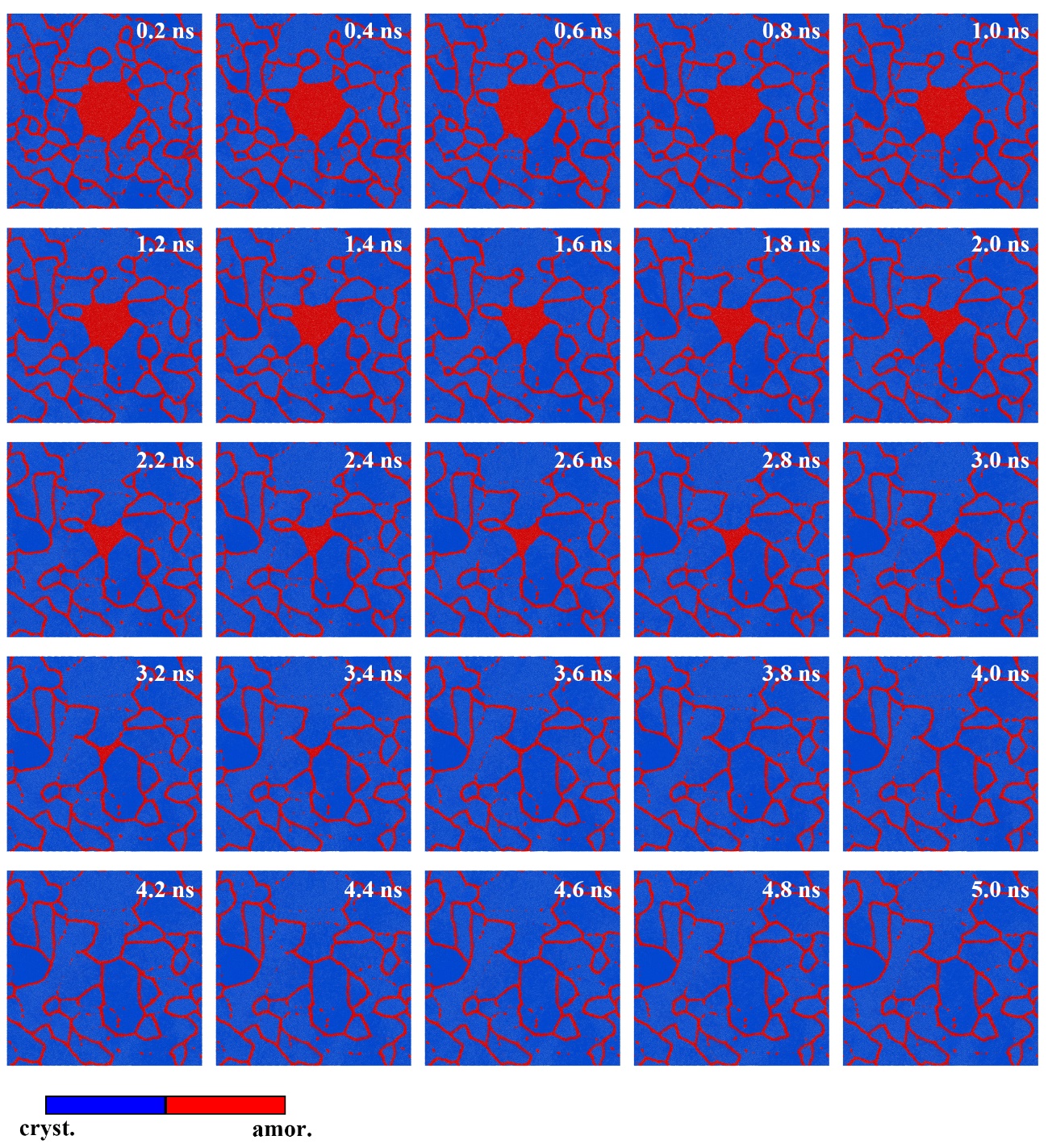


**Fig. S16** Simulated crystallization process of amorphous Sb film


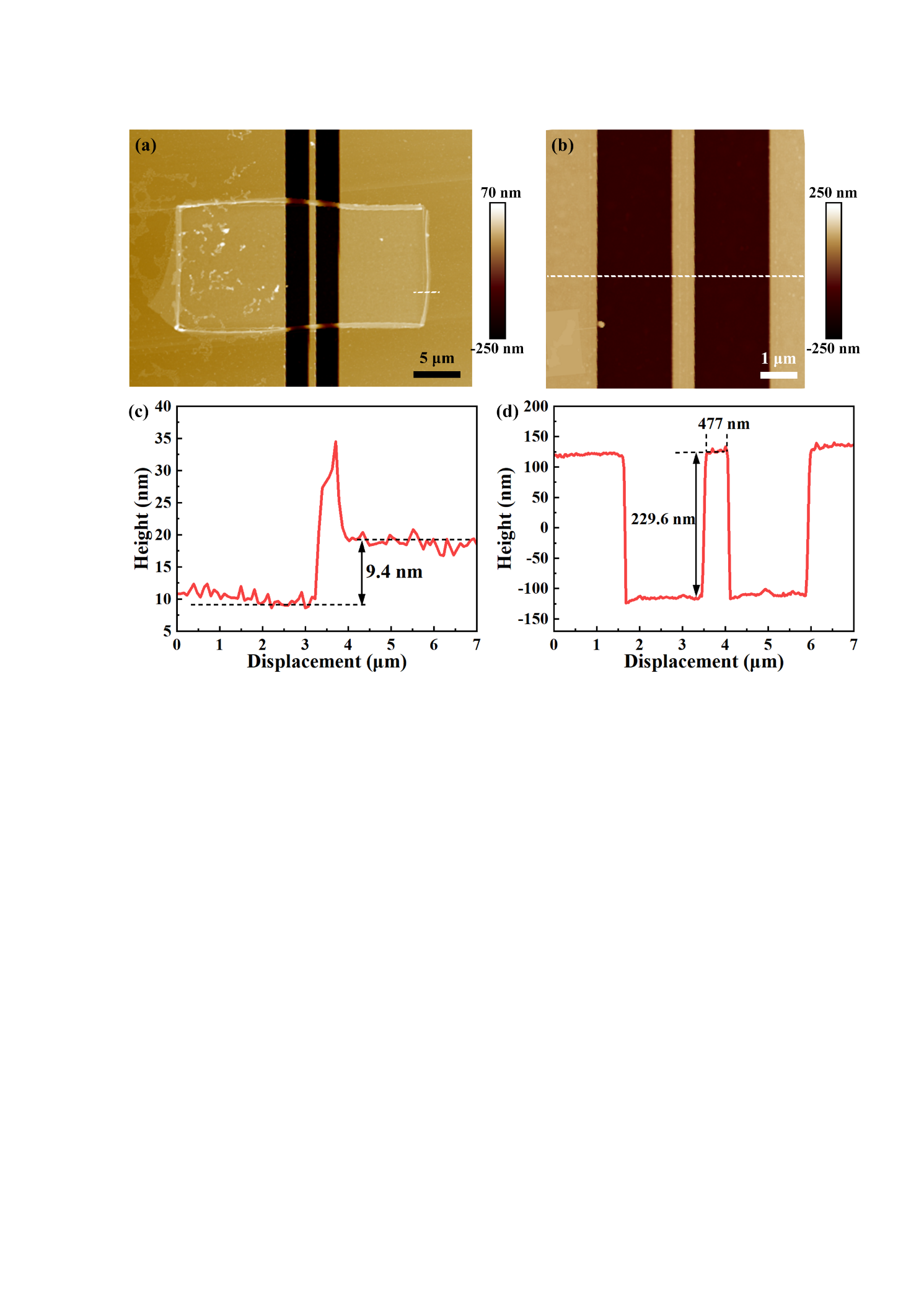


**Fig. S17** AFM characterization of fabricated waveguide. (**a**) The two-dimensional mapping of waveguide. (**b**) The enlarged view of a. (**c, d**) The sectional profile along the dashed line in a and b.





**Fig. S18** Numerical testing results of accuracy and loss versus epoch number


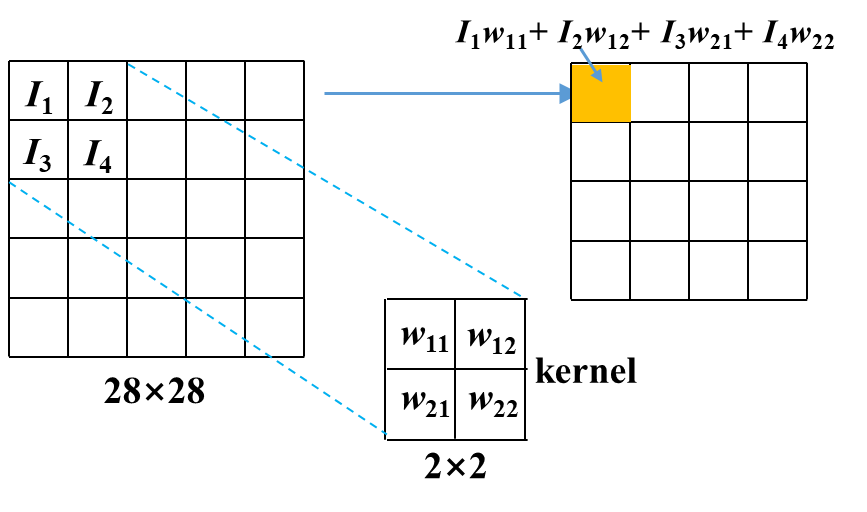


**Fig. S19** Working principle of photonic convolutional kernel


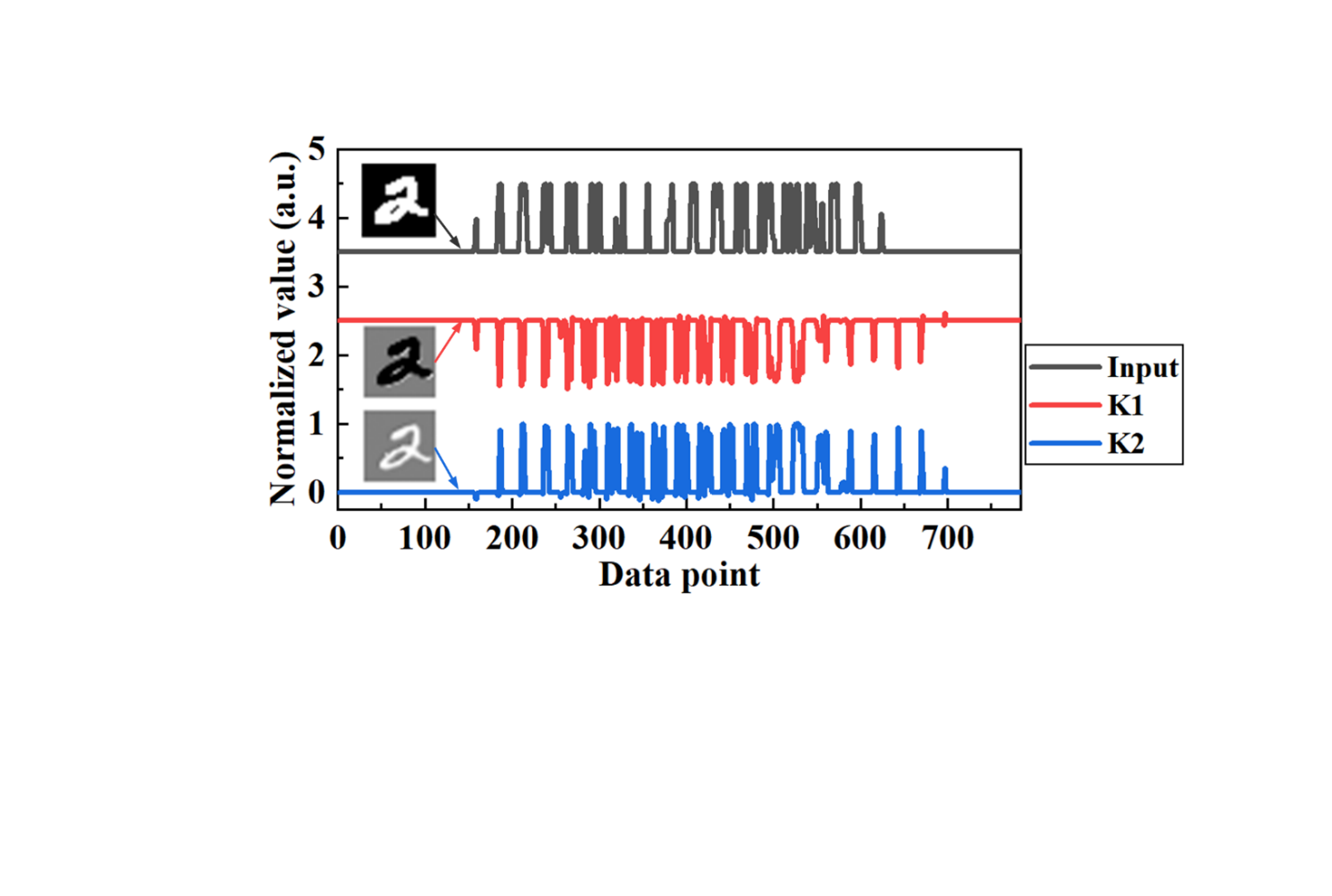


**Fig. S20** Input and output signals of number “2”
